# Hepatic non-parenchymal S100A9-TLR4-mTORC1 axis normalizes diabetic ketogenesis

**DOI:** 10.1038/s41467-022-31803-5

**Published:** 2022-07-15

**Authors:** Gloria Ursino, Giorgio Ramadori, Anna Höfler, Soline Odouard, Pryscila D. S. Teixeira, Florian Visentin, Christelle Veyrat-Durebex, Giulia Lucibello, Raquel Firnkes, Serena Ricci, Claudia R. Vianna, Lin Jia, Mirjam Dirlewanger, Philippe Klee, Joel K. Elmquist, Johannes Roth, Thomas Vogl, Valérie M. Schwitzgebel, François R. Jornayvaz, Andreas Boland, Roberto Coppari

**Affiliations:** 1 Department of Cell Physiology and Metabolism, University of Geneva, 1211 Geneva, Switzerland; 2 Diabetes Center of the Faculty of Medicine, University of Geneva, 1211 Geneva, Switzerland; 3 Department of Molecular Biology, University of Geneva, 1211 Geneva, Switzerland; 4 Center for Hypothalamic Research, Department of Internal Medicine, University of Texas Southwestern Medical Center at Dallas, Dallas, TX 75390 USA; 5 Pediatric Endocrine and Diabetes Unit, Department of Pediatrics, Obstetrics and Gynecology, University Hospitals of Geneva, Geneva, Switzerland; 6 Department of Pharmacology, University of Texas Southwestern Medical Center at Dallas, Dallas, TX 75390 USA; 7 Institute of Immunology, University of Munster, 48149 Munster, Germany; 8 Interdisciplinary Centre for Clinical Research, University of Munster, 48149 Munster, Germany; 9 Service of Endocrinology, Diabetes, Nutrition and Therapeutic patient education, Geneva University Hospitals, 1205 Geneva, Switzerland

**Keywords:** Translational research, Type 1 diabetes, Recombinant protein therapy

## Abstract

Unrestrained ketogenesis leads to life-threatening ketoacidosis whose incidence is high in patients with diabetes. While insulin therapy reduces ketogenesis this approach is sub-optimal. Here, we report an insulin-independent pathway able to normalize diabetic ketogenesis. By generating insulin deficient male mice lacking or re-expressing Toll-Like Receptor 4 (TLR4) only in liver or hepatocytes, we demonstrate that hepatic TLR4 in non-parenchymal cells mediates the ketogenesis-suppressing action of S100A9. Mechanistically, S100A9 acts extracellularly to activate the mechanistic target of rapamycin complex 1 (mTORC1) in a TLR4-dependent manner. Accordingly, hepatic-restricted but not hepatocyte-restricted loss of Tuberous Sclerosis Complex 1 (TSC1, an mTORC1 inhibitor) corrects insulin-deficiency-induced hyperketonemia. Therapeutically, recombinant S100A9 administration restrains ketogenesis and improves hyperglycemia without causing hypoglycemia in diabetic mice. Also, circulating S100A9 in patients with ketoacidosis is only marginally increased hence unveiling a window of opportunity to pharmacologically augment S100A9 for preventing unrestrained ketogenesis. In summary, our findings reveal the hepatic S100A9-TLR4-mTORC1 axis in non-parenchymal cells as a promising therapeutic target for restraining diabetic ketogenesis.

## Abstract (web summary)

Excess ketogenesis can lead to ketoacidosis, a serious complication in patients with diabetes. Here the authors report an insulin independent pathway, the hepatic nonparenchymal S100A9-TLR4-mTORC1 axis, that is able to normalize diabetic ketogenesis and pre-clinical data to suggest potential for development of S100A9 based adjunctive therapy to insulin.

## Introduction

Physiological ketogenesis is an adaptive mechanism leading to increased production of ketone bodies (i.e., acetoacetate, acetone, and β-hydroxybutyrate); a process allowing mammals to survive during prolonged fasting^1,2^. Ketogenesis mainly occurs in the mitochondria of hepatocytes where, by a series of biochemical reactions mediated by acetoacetyl-CoA thiolase (ACAT1), 3-hydroxy-3-methylglytaryl-CoA synthetase (HMGCS2; the rate-limiting enzyme), HMG-CoA lyase (HMGCL), and β-hydroxybutyrate dehydrogenase (BDH1), acetyl-CoA is used as substrate for the production of acetoacetate and β-hydroxybutyrate^2–5^. Hepatocytes secrete acetoacetate and β-hydroxybutyrate into the circulation from where they are taken up via monocarboxylate transporter 1 (MCT1) and are oxidized to generate ATP in virtually all cells but hepatocytes. Indeed, the ketolysis program requires the action of mitochondrial succinyl-CoA:3-ketoacid-coenzyme A transferase (SCOT); an enzyme present in every cell but hepatocytes^1,2^. Acetone is produced by spontaneous decarboxylation of acetoacetate in some tissues as for example the lung; hence explaining the characteristic smell of acetone in the breath of people suffering from heightened ketogenesis^3^.

The regulation of physiological ketogenesis is due but not limited to changes in endocrine function. For example, during a period of prolonged starvation the circulating level of insulin is significantly reduced. As insulin is a potent inhibitor of lipolysis and proteolysis, in this context the rate of lipolysis in adipose tissue is exacerbated leading to increased release of free fatty acids (FFAs). These are taken up by hepatocytes where they undergo β-oxidation to generate acetyl-CoA^6^. In addition, enhanced proteolysis fuels ketogenesis via catabolism of ketogenic amino acids^7^. Insulin is also a potent inhibitor of gluconeogenesis. Therefore, in the fasted state the increased rate of gluconeogenesis prevents oxaloacetate (that is highly used for gluconeogenesis) to undergo condensation with acetyl-CoA to form citrate. Concomitantly, starvation leads to increased secretion of glucagon that stimulates hepatic fatty acids oxidation (FAO)^8^. Overall, the aforementioned hormonal changes contribute to an overabundance of mitochondrial acetyl-CoA that is used for ketogenesis^2–5^. Mechanistically, increased hepatic Sirtuin 3 (SIRT3), forkhead box protein A2 (FOXA2), peroxisome proliferator activated receptor alpha (PPARα) and AMP-activated protein kinase (AMPK) activities stimulate, while increased AKT and mTORC1 pathways dampen ketogenesis^9–16^. In addition to the aforementioned intra-hepatic mechanisms, extra-hepatic pathways have recently been reported. For example, the increased histamine release from mast cells leads to oleoylethanolamide-mediated activation of hepatic PPARα; an effect contributing to fasting-induced ketogenesis^17^. In summary, physiological ketogenesis is a life-protecting adaptive mechanism aimed at providing ketone bodies as source of energy for the whole body (except for hepatocytes) during periods of prolonged starvation^18^.

Pathological ketogenesis is, on the other hand, life-threatening. For example, diabetic hyperketonemia develops when circulating insulin levels are insufficient to suppress ketogenesis hence leading to uncontrolled ketone body production. If severe, this defect brings about diabetic ketoacidosis (DKA; that is blood acidification owing to the low acid dissociation constant of ketone bodies) that can be fatal; therefore, DKA needs emergency medical attention^19^. DKA mainly involves subjects with insulin deficiency (ID); a condition affecting tens of millions worldwide^20,21^. These include i) all people who underwent complete pancreatectomy, ii) all subjects suffering from type 1 diabetes (T1D; a condition in which insulin-producing pancreatic β-cells are destroyed)^22^, and iii) a significant percentage of subjects affected by type 2 diabetes (T2D; a disease in which pancreatic β-cells can undergo exhaustion, failure, and/or dedifferentiation)^20,21^. While insulin therapy is pivotal to reduce ketogenesis in these subjects this approach is sub-optimal. Indeed, patients with diabetes are at a much higher risk of developing ketoacidosis as compared to healthy subjects^19,23,24^. For example, in 2014 in the United States of America there were 44.3 DKA hospitalizations for each 1000 patients with diabetes who were 45-years-old or younger^25^. Moreover, analysis of several adult T1D patient populations in different countries between 2000 and 2016 reported an even higher incidence of DKA hospitalization (e.g., with a range reaching 263 cases for each 1000 patients per year)^26^. Based on the aforementioned reports, the prevalence of DKA hospitalization per year in T1D patients is in the range of 2–4%. Therefore, identifying measure(s) able to restrain ketogenesis in patients with poorly controlled diabetes is of major medical importance.

Mechanistically, little is known about the pathways underlying diabetic ketogenesis. While insulin-dependent mechanisms are clearly involved, the direct action of insulin on hepatocytes appears irrelevant as mice lacking the insulin receptor only in hepatocytes do not display increased ketogenesis^27^. Of note, recent studies revealed the existence of insulin-independent mechanisms that can also normalize diabetic ketogenesis. The seminal studies by the Unger group demonstrated that leptin monotherapy normalizes the hyperketonemia (and several other metabolic defects) caused by ID in rodents^28,29^. Following studies revealed that the beneficial effect of leptin is mediated by the brain^30–32^ and more specifically by a subset of hypothalamic γ-aminobutyric acid (GABA)-ergic and pro-opiomelanocortin (POMC) neurons^33^. By acting on these neurons, leptin augments the content of circulating S100 calcium-binding protein A9 (S100A9). S100A9 belongs to the EF-hand superfamily of Ca^2+^-binding proteins and forms heterocomplexes with another protein family member (S100A8) but also homodimerizes, which has been reported to directly affect Toll-like receptor 4 (TLR4) signaling^34–36^.

TLR4 recognizes lipopolysaccharide (LPS), as well as several viral proteins, polysaccharide and a variety of endogenous proteins such as low-density lipoprotein, beta-defensins, and heat shock proteins^37^. Activation of TLR4 at the cell surface leads to dimerization and recruitment of its adaptor proteins Myeloid Differentiation Primary Response Gene 88 (MyD88) and TIR Domain-Containing Adaptor Protein (TIRAP) which activate a signaling cascade resulting in the activation of transcription factors, such as Nuclear Factor Kappa Beta (NFκB) and Activator Protein-1 (AP-1) for the production of inflammatory cytokines^38^. TLR4 signaling also involves the activation of the MyD88-independent or TRIF TRAM pathway, which involves recruitment of the adaptors TIR-domain-containing adaptor inducing interferon-β (TRIF) and TRIF-related Adaptor Molecule (TRAM). TRAM-TRIF signals activate the transcription factor Interferon Regulatory Factor-3 (IRF3) via TRAF3. IRF3 activation induces the production of type 1 interferons^39^. Excess activation of MyD88 and TRIF-TRAM pathways has been associated with septic shock^40^. However, surface activation of TLR4 also invokes the phosphatidylinositol 3-kinase (PI3K)/Akt pathway which has been shown to bias the cytokine response away from a hyperinflammatory setting by decreasing the production of pro-inflammatory cytokines and increasing the production of anti-inflammatory cytokines^41^.

Despite its predicted pro-inflammatory function, overexpression of S100A9 has recently been shown to exert hyperketonemia-normalizing and pro-survival action via TLR4-dependent mechanisms in ID mice^42^. Thus, harnessing this insulin-independent mechanism (in combination with insulin therapy) is anticipated to reduce DKA incidence and improve management of diabetes. Yet, this mechanism and its therapeutic potential still remains poorly understood. For example, i) in which tissue(s) TLR4 mediates the beneficial action of S100A9, ii) whether this action is due to extracellular and/or intracellular S100A9, iii) the identity of intracellular pathways involved, and most importantly from a clinical point of view iv) whether S100A9 is therapeutically suitable is unknown.

In this study, by generating and assessing diverse genetically engineered animal models of diabetic hyperketonemia we determined the site (i.e., liver) and key molecular and cellular components (i.e., TLR4 and mTORC1 in non-parenchymal hepatic cells) underlying the hyperketonemia-normalizing effect of S100A9. Furthermore, we show pre-clinical and observational clinical data indicating that recombinant S100A9 is a putative investigational drug displaying safety and efficacy profiles owning therapeutic potential.

## Results

### S100A9 suppresses diabetic ketogenesis via hepatic TLR4

The mechanism(s) underlying the beneficial action of S100A9 in insulin deficiency (ID) is poorly understood^42^. As the circulating level of the heterodimer S100A9/S100A8 (calprotectin) is increased in ID and reduced by S100A9^42^, we investigated the possibility that lowered calprotectin content improves metabolic imbalance in ID. To directly test this assumption, we generated mice lacking S100A9 and are therefore unable to produce S100A8 and consequently, calprotectin^36^. By crossing the *RIP-DTR* allele [this allele bears a rat insulin promoter (RIP) upstream of diphtheria toxin receptor (DTR) sequences^43^] with the *S100a9* null allele^36^ we obtained calprotectin-deficient *RIP-DTR*; *S100a9*^*-/-*^ mice and their calprotectin-intact *RIP-DTR*; *S100a9*^*+/+*^ littermate controls. *RIP-DTR*; *S100a9*^*-/-*^ and *RIP-DTR*; *S100a9*^*+/+*^ mice displayed similar levels of circulating glucose, insulin, triglycerides, and β-hydroxybutyrate (the most abundant ketone body in the blood)^3^ and had undistinguishable body weight (Supplementary Fig. 1A–E). These data demonstrate that calprotectin deficiency does not affect metabolic homeostasis. Following three consecutive intraperitoneal DT administrations, *RIP-DTR* mice develop a near-total-loss of pancreatic β-cells and display the key clinical symptoms of ID^33^. Accordingly, DT-treated *RIP-DTR*; *S100a9*^*+/+*^ mice acquired severe hypoinsulinemia, hyperketonemia, hyperglycemia, hypertriglyceridemia and reduced body weight compared to their DT-untreated *RIP-DTR*; *S100a9*^*+/+*^ healthy controls (Supplementary Fig. 1A–E). A similar degree of hypoinsulinemia, hyperketonemia, hyperglycemia, hypertriglyceridemia and reduced body weight was observed in DT-treated *RIP-DTR*; *S100a9*^*-/-*^ mice compared to DT-treated *RIP-DTR*; *S100a9*^*+/+*^ controls (Supplementary Fig. 1A–E). Thus, our data refute the assumption that lowered calprotectin content improves hyperketonemia and metabolic imbalance caused by ID.

**Fig. 1:**
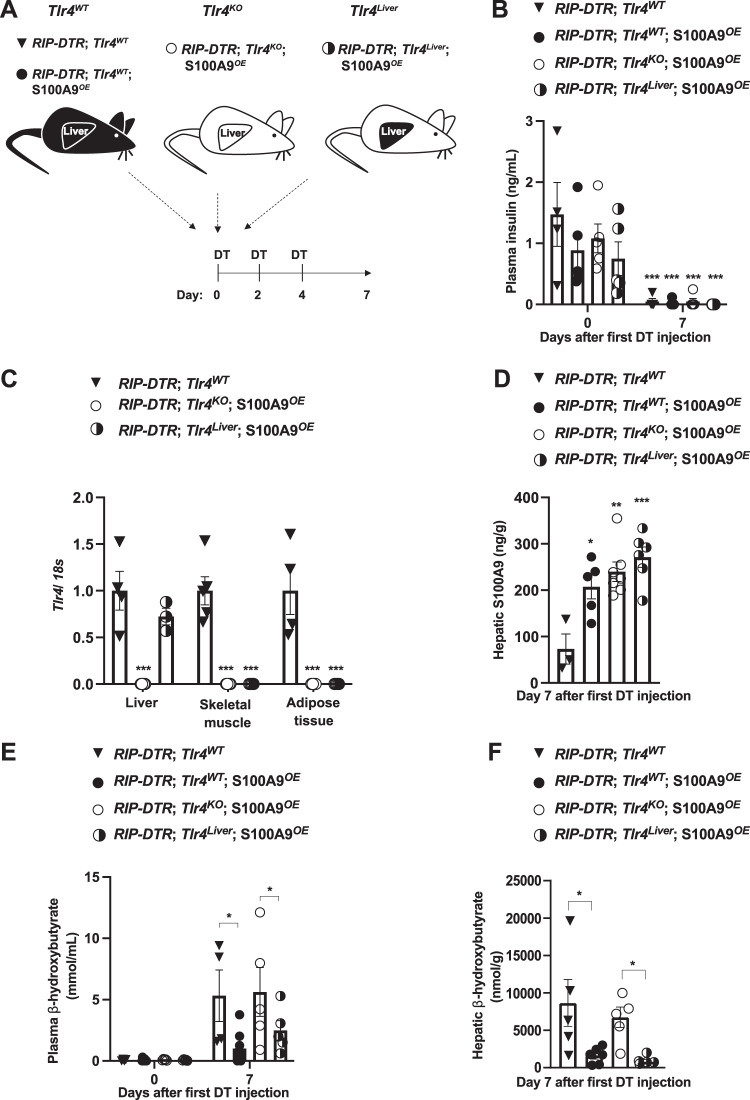
S100A9 suppresses diabetic ketogenesis via hepatic TLR4. **A** Scheme indicating the generation of RIP-DTR;*Tlr4*^*WT*^ (*n* = 5), RIP-DTR;*Tlr4*^*WT*^; S100A9^*OE*^ (*n* = 6), RIP-DTR;*Tlr4*^*KO*^; S100A9^*OE*^ (*n* = 7) and RIP-DTR;*Tlr4*^*Liver*^; S100A9^*OE*^ (*n* = 6) experimental groups. Adenoviral injections and HTVI were done 3 days before or the same day of the first DT injection, respectively. **B** Plasma insulin levels of mice at day 0 (i.e., before DT injection) and 7 days after first DT injection (*p* = 0.001). **C** mRNA content of *Tlr4* in the liver, muscle (gastrocnemius), and adipose tissue (interscapular brown adipose tissue) of indicated groups (*p* = 0.001). **D** Hepatic S100A9 content of indicated cohorts 7 days after first DT injection (*p* = 0.011, *p* = 0.001 & *p* = 0.0003). **E** Plasmatic hepatic β-hydroxybutyrate levels in the indicated cohorts and time after first DT injection (*p* = 0.017 and *p* = 0.012) and **F** hepatic β-hydroxybutyrate levels in the indicated cohorts and time after first DT injection (*p* = 0.03, *p* = 0.003). Error bars represent SEM, statistical analyses were done using one-way or two-way ANOVA (Tukey’s post- hoc test). In **B**, comparison was made to basal values. In **C**–**F** comparison was made to RIP-DTR; *Tlr4*^WT^ group or otherwise indicated. *p ≤ 0.05, **p ≤ 0.01, ***p ≤ 0.001. Source data are provided as a source data file.

The hyperketonemia-normalizing action of S100A9 requires TLR4^42^. Yet, in which tissue(s)/cell type(s) TLR4 mediates the beneficial action of S100A9 is unknown. Considering the key role of the liver in ketogenesis^3^, we directly tested the relevance of hepatic TLR4. The generation of ID mice selectively expressing TLR4 in liver and concomitantly overexpressing S100A9 was achieved as follows. To accomplish hepatic-specific TLR4 expression we took advantage of a genetically engineered *Tlr4*^*LoxTB*^ allele; this is a Cre-recombinase-reactivable *Tlr4* null allele allowing restoration of the endogenous TLR4 expression in any Cre-recombinase-expressing cell type(s) (Supplementary Fig. 1F)^44^. Further confirming that the *Tlr4*^*LoxTB*^ allele is a *Tlr4* null allele^44^, administration of lipopolysaccharides (LPS; a TLR4 ligand known to activate TLR4 signaling) caused a significant increase in the level of circulating tumor necrosis factor alpha (TNF-α) in mice homozygous for the wild-type *Tlr4* allele (*Tlr4*^*WT*^ mice); yet, this effect was lost in mice homozygous for the *Tlr4*^*LoxTB*^ allele (referred to as *Tlr4*^*KO*^ mice) (Supplementary Fig. 1G). To achieve ID, we exploited the *RIP-DTR* allele^43^. S100A9 overexpression was attained by hydrodynamic tail vein injection (HTVI) of a plasmid bearing *S100a9* coding sequences under the control of the albumin promoter (pLIVE-S100A9) as previously described^42^. Mice homozygous for the *Tlr4*^*LoxTB*^ allele and carrying the *RIP-DTR* allele were treated with DT, infected with Adenovirus-Cre-GFP serotype 5 (that has a high degree of hepatotropism, infecting both hepatocytes and other non-parenchymal hepatic cells (e.g., F480 + expressing macrophages Supplementary Fig. 1H, I)^45^, and underwent HTVI of pLIVE-S100A9 (referred to as *RIP-DTR*; *Tlr4*^*liver*^; S100A9^*OE*^ mice) as indicated in Fig. 1A. As controls, ID mice lacking TLR4 and overexpressing S100A9 were generated. These mice were homozygous for the *Tlr4*^*LoxTB*^ allele, bore the *RIP-DTR* allele, were treated with DT, infected with Adenovirus-GFP serotype 5, and underwent HTVI of pLIVE-S100A9 (referred to as *RIP-DTR*; *Tlr4*^*KO*^; S100A9^*OE*^ mice) as indicated in Fig. 1A. Two additional control groups of ID mice endogenously expressing TLR4 and either overexpressing or endogenously expressing S100A9 were also generated. These mice were homozygous for the wild-type *Tlr4* allele, bore the *RIP-DTR* allele, were treated with DT, infected with Adenovirus-GFP serotype 5, and underwent HTVI of either pLIVE (referred to as *RIP-DTR*; *Tlr4*^*WT*^ mice) or pLIVE-S100A9 (referred to as *RIP-DTR*; *Tlr4*^*WT*^; S100A9^*OE*^ mice) as indicated in Fig. 1A.

Following DT administration, all the aforementioned groups developed a similar degree of ID (Fig. 1B). *RIP-DTR*; *Tlr4*^*KO*^; S100A9^*OE*^ mice displayed a remarkable reduction in *Tlr4* mRNA content in several metabolically relevant tissues compared to their TLR4-intact controls (*RIP-DTR*; *Tlr4*^*WT*^ mice) (Fig. 1C). Importantly, while *Tlr4* mRNA level remained dramatically reduced in skeletal muscle and adipose tissue it was normalized in the liver of *RIP-DTR*; *Tlr4*^*liver*^; S100A9^*OE*^ mice (Fig. 1C). These data are in line with the robust GFP expression that was observed in approximately 93% of cells in hepatic tissue from infected mice (Supplementary Fig. 1H). S100A9 levels were increased in *RIP-DTR*; *Tlr4*^*KO*^; S100A9^*OE*^, and *RIP-DTR*; *Tlr4*^*WT*^; S100A9^*OE*^ mice as well as in *RIP-DTR*; *Tlr4*^*liver*^; S100A9^*OE*^ mice as compared to their *RIP-DTR*; *Tlr4*^*WT*^ controls (Fig. 1D). Jointly, these results validate our animal models.

ID causes hyperketonemia, a defect that is improved by S100A9 overexpression^42^. Indeed, while circulating β-hydroxybutyrate content was significantly increased in DT-treated *RIP-DTR*; *Tlr4*^*WT*^ mice compared to their basal (i.e., before DT injection) level, this defect was greatly improved by S100A9 overexpression as circulating β-hydroxybutyrate content was significantly reduced in DT-treated *RIP-DTR*; *Tlr4*^*WT*^; S100A9^*OE*^ mice compared to DT-treated *RIP-DTR*; *Tlr4*^*WT*^ mice (Fig. 1E). Underscoring the importance of TLR4, circulating β-hydroxybutyrate level remained elevated in DT-treated *RIP-DTR*; *Tlr4*^*KO*^; S100A9^*OE*^ mice (Fig. 1E). Noteworthy, the ketogenesis-lowering action of S100A9 was restored by selective expression of TLR4 in the liver because DT-treated *RIP-DTR*; *Tlr4*^*liver*^; S100A9^*OE*^ mice exhibited similar circulating β-hydroxybutyrate content as DT-treated *RIP-DTR*; *Tlr4*^*WT*^; S100A9^*OE*^ mice (Fig. 1E). Furthermore, while hepatic content of β-hydroxybutyrate was elevated in DT-treated *RIP-DTR*; *Tlr4*^*WT*^ and *RIP-DTR*; *Tlr4*^*KO*^; S100A9^*OE*^ mice it was significantly and similarly reduced in DT-treated *RIP-DTR*; *Tlr4*^*WT*^; S100A9^*OE*^ and *RIP-DTR*; *Tlr4*^*liver*^; S100A9^*OE*^ mice (Fig. 1F). Circulating non-esterified fatty acids (NEFAs) were all increased compared to basal levels in all groups after induction of ID, but no change was observed between groups after DT treatment (Supplementary Fig. 1J). Similarly, no change in adipose tissue lipase activity was observed between ID groups (Supplementary Fig. 1K). Collectively, these data demonstrate that hepatic TLR4 is sufficient for mediating the ketogenesis-lowering action of S100A9 in diabetic mice.

### S100A9 suppresses diabetic ketogenesis by activating hepatic mTORC1 via TLR4

The aforementioned data revealed a key role of hepatic S100A9-TLR4 axis in suppressing pathological ketogenesis; yet, the identity of the downstream intracellular pathway(s) involved is unknown. Because the mechanism(s) driving diabetic ketogenesis is poorly understood, we started by examining signaling pathways that regulate physiological ketogenesis. Fasting-induced ketogenesis is achieved, at least in part, by activation of hepatic AMPK and glucagon receptor signaling^3,10^. Owing to their ID, *RIP-DTR*; *Tlr4*^*WT*^ mice displayed increased level of hepatic phosphorylated acetyl-CoA carboxylase (pACC; an established readout of AMPK activity)^46^ and phosphorylated CREB (pCREB, an established readout of glucagon receptor signaling)^30^ compared to their healthy controls (Supplementary Fig. 2A–C). However, neither of these pathways were affected by S100A9 overexpression as hepatic contents of pACC/ACC and pCREB/CREB were not different between ID; *RIP-DTR*; *Tlr4*^*WT*^ and ID; *RIP-DTR*; *Tlr4*^*WT*^; S100A9^*OE*^ mice (Supplementary Fig. 2A–C).

**Fig. 2:**
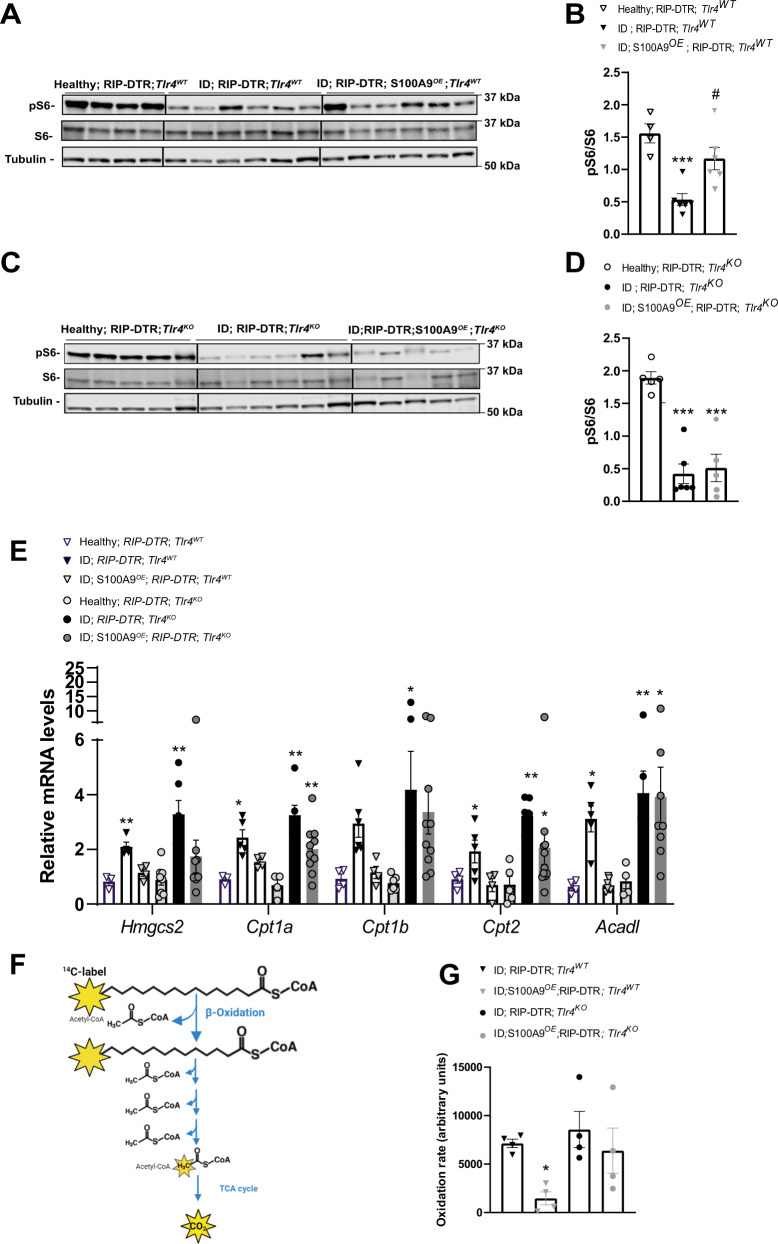
S100A9 overexpression rescues hepatic mTORC1 signaling and dampens ketogenic gene expression. **A** RIP-DTR; *Tlr4*^*WT*^ mice were made insulin deficient with DT and underwent HTVI with 50ug of either a plasmid encoding for mouse S100A9 (RIP-DTR; S100A9^*OE*^) (*n* = 6) or empty vector (*n* = 6). 7 days after the first DT injection these mice were fasted for 3 h and sacrificed along with healthy controls (*n* = 4) and liver lysates were analyzed by immunoblotting for the indicated proteins and phosphorylation states. **B** Densitometry of pS6/S6 ratio of immunoblot from **A** (*p* = 0.009 and *p* = 0.01). **C** RIP- DTR; *Tlr4*^*KO*^ mice were made insulin deficient with DT and underwent HTVI of 50 ug of either a plasmid encoding for mouse S100A9 (RIP-DTR; S100A9^*OE*^) (*n* = 5) or empty vector (*n* = 6). 7 days after first DT injection mice were fasted for 3 h and liver lysates from these and healthy controls (*n* = 5) were analysed by immunoblotting for the indicated proteins and phosphorylation states **D** Densitometry of pS6/S6 ratio of immunoblot from (*p* = 0.0001) **C**. **E** qRT-PCR analysis of genes from liver of *Tlr4*^*W*T^ healthy, ID (7 days after the first DT injection) and ID S100A^*OE*^ groups (*n*/group = 4, 5 and 4) and *Tlr4*^*KO*^ healthy, ID and ID;S100A9^*OE*^ groups (n/group = 7, 6 and 8) indicated. Values are relative to healthy RIP-DTR; *Tlr4*^*WT*^ mice (*Hmgcs2:* *P* = 0.005, 0.002; *Cpt1a* *p* = 0.04, *p* = 0.001, *p* = 0.001; *Cpt2* *p* = 0.02, *p* = 0.0026, *p* = 0.0314, *Acadl* *p* = 0.03, *p* = 0.0021, *p* = 0.0324). **F** Schematic overview of metabolic 1-^14^C-palmitic acid tracing assay. **G** Hepatic fatty acid oxidation rate (using 1-^14^C-palmitic acid) (*n*/group = 4, 5, 4 and 4) (*p* = 0.0517). Error bars represent SEM, statistical analyses were done using one way or two-way ANOVA (Tukey’s post-hoc test). In **B**, **D**, * and **#** indicate comparison to healthy group and between ID groups, respectively. *P < 0.05, **P < 0.01, ***P < 0.01 ^#^*P* < 0.05, ^##^*P* < 0.01, ^###^*P* < 0.001. Source data are provided as a source data file.

Based on the knowledge that i) TLR4 is required and sufficient for the hyperketonemia-normalizing action of S100A9 (Fig. 1E, F), ii) TLR4 activates the mTOR axis downstream of the TRIF/TRAM pathway^41,47,48^, and iii) mTORC1 suppresses fasting-induced hyperketonemia^10^, we investigated whether mTORC1 is a key molecular component of the pathway(s) underling the hyperketonemia-normalizing action of S100A9 in diabetes. First, we assessed the hepatic level of phosphorylated ribosomal S6 protein and phosphorylated 4E-BP1 (established markers of mTORC1 activity^10,49^)in ID; *RIP-DTR*; *Tlr4*^*WT*^ mice and found them to be significantly reduced compared to their healthy controls (Fig. 2A, B and Supplementary Fig. 2D). Second, we tested whether S100A9 overexpression is able to rescue this defect. Our data shown in Fig. 2A, B indicate that *RIP-DTR*; *Tlr4*^*WT*^; S100A9^*OE*^ mice have increased level of hepatic pS6/S6 as compared to *RIP-DTR*; *Tlr4*^*WT*^; also, the level of hepatic pS6/S6 in *RIP-DTR*; *Tlr4*^*WT*^; S100A9^*OE*^ mice is similar to their healthy controls. Third, to test whether S100A9-induced activation of hepatic mTORC1 activity requires TLR4 we analyzed ID mice lacking TLR4 with or without S100A9 overexpression (*RIP-DTR*; *Tlr4*^*KO*^; S100A9^*OE*^ and *RIP-DTR*; *Tlr4*^*KO*^ mice, respectively). In the context of TLR4 deficiency, ID also led to reduced hepatic pS6/S6 content; yet, in contrast to the TLR4-intact context S100A9 overexpression was unable to correct this defect as the level of hepatic pS6/S6 was similarly reduced in *RIP-DTR*; *Tlr4*^*KO*^; S100A9^*OE*^ and *RIP-DTR*; *Tlr4*^*KO*^ mice compared to their healthy controls (Fig. 2C, D). Similar results were also obtained for p-4E-BP1 (Supplementary Fig. 2D, E). Importantly, levels of hepatic pS6/S6 content were not changed between *Tlr4*^*WT*^ and *Tlr4*^*KO*^ in both healthy and ID context (Supplementary Fig. 2F, G). Next, we investigated hepatic activation of key signaling pathways upstream of mTORC1, the AKT and the extracellular-regulated kinase (ERK) signaling by assessing their phosphorylated status^50^. Of note, the level of pAKT/AKT was decreased in ID mice in both genotypes compared to healthy controls and rescued by S100A9^*OE*^ only in *Tlr4*^*WT*^ mice (Supplementary Fig. 2A, Supplementary Fig. 2H and Supplementary Fig. 2I). p-ERK levels were decreased in ID but not changed by S100A9^*OE*^ (Supplementary Fig. 2J). Finally, there was no correlation between levels of hepatic mTORC1 activation and plasma ketones or hepatic pAKT signaling; this phenomenon could be due to the fact that i) these parameters are unrelated to one another or ii) the time at which these parameters have been measured was not appropriate to unmask correlation (Supplementary Fig. 2K, L). Overall, these data suggest that S100A9 activates hepatic mTORC1 in a TLR4-dependent fashion.

Due to the fact that S100A9 activates TLR4, we examined read-outs of the TLR4-NFκΒ signaling pathway. Compared to healthy controls, ID mice displayed increased circulating TNFα and increased hepatic *Tnfa* and *Ikba* mRNA level (Supplementary Fig. 2M–O). Surprisingly, ID S100A9^*OE*^ mice show a reduction rather than an increase in these parameters (Supplementary Fig. 2M–O), indicating TLR4 signaling is activated to cause a net anti-inflammatory rather than pro-inflammatory response. Similarly, compared to LPS treated positive controls we failed to see a significant increase in other hepatic canonical TLR4 read-outs such as NF-Κβ and INF-1β (Supplementary Fig. 2P–R).

Circulating NEFAs level were increased in ID mice compared to healthy controls, but no change was observed between groups after induction of ID (Supplementary Fig. 2S). Therefore, we directly measured genes involved in hepatic fatty acid oxidation (FAO).

mTORC1 suppresses fasting-induced hyperketonemia via inhibition of the nuclear hormone receptor PPARα, a master regulator of genes involved in ketogenesis and FAO^10^. Thus, in agreement with reduced hepatic mTORC1 signaling (Fig. 2A, B) ID mice displayed increased mRNA level of key PPARα’s target genes involved in hepatic FAO and ketogenesis as for example *Hmgcs2*, *Cpt1a*, *Cpt1b, Cpt2* and *Acadl* (Fig. 2E, *RIP-DTR*; *Tlr4*^*WT*^ vs. their healthy controls). Noteworthy, these defects were partially corrected by S100A9 overexpression (Fig. 2E, *RIP-DTR*; *Tlr4*^*WT*^; S100A9^*OE*^ vs. *RIP-DTR*; *Tlr4*^*WT*^ and their healthy controls). Nevertheless, the ability of S100A9 to reduce expression of the aforementioned PPARα’s target genes is lost in the context of TLR4 deficiency (Fig. 2E). To directly test whether the abovementioned changes in PPARα’s target genes led to any biological consequence, we directly measured the rate of hepatic FAO with radioactive tracing of ^14^C palmitate (Fig. 2F). In line with the changes in PPARα’s target genes, S100A9 dampened hepatic FAO in *Tlr4*^*WT*^ but failed to mediate this effect in *Tlr4*^*KO*^ ID mice (Fig. 2G). Hence, the hepatic-FAO-suppressive effect of S100A9 requires TLR4.

**Fig. 3:**
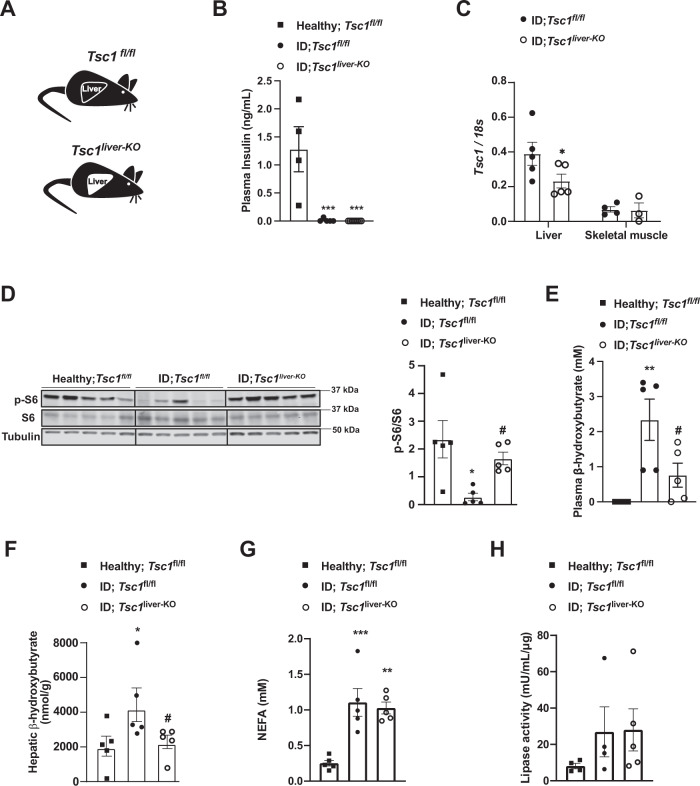
Liver specific up-regulation of mTORC1 signaling prevents hyperketonemia in ID. **A** Experimental groups. Homozygous Tuberous sclerosis complex 1 floxed (*Tsc1*^*fl/fl*^) mice were administered 1 × 10^9 PFU Adenovirus serotype 5 expressing Cre and GFP (Cre recombinantion results in a *Tsc1* null allele in the liver of *Tsc1*^*fl/fl*^ mice): *Tsc1*^liver-KO^. Control mice were treated with 1 × 10^9 PFU Adenovirus serotype 5 expressing only GFP: *Tsc1*^*fl/fl*^. **B** Plasma insulin levels of insulin deficient mice and healthy controls (*p* = 0.0003 and *p* = 0.0001). **C** mRNA content of *Tcs1* in the liver (*p* = 0.0584) and skeletal muscle (gastrocnemius) of the indicated groups. **D** Image of immunoblot for the indicated proteins and phosphorylation states and relative densitometry quantification of pS6/S6 (*p* = 0.011, *p* = 0.05). **E** Plasmatic β-hydroxybutyrate levels of indicated groups (*p* = 0.008, *p* = 0.048). **G** Plasma NEFAs level (*p* = 0.008, *p* = 0.002). **H** Lipase activity from perigonadal fat. (*n*/group = 5, 5 and 5) Error bars represent SEM. Statistical analyses were done using one-way ANOVA (Tukey’s post-hoc test, except for **F** in which FDR was used) (* and # indicate comparison to healthy and to control mice, respectively), except for **C** for which analysis were done using a two- tailed unpaired Student’s t-test. *P < 0.05, **P < 0.01, ***P < 0.001, ^#^*P* ≤ 0.05, ^##^*P* ≤ 0.01. Western blot images shown in **D** are cropped images. Source data are provided as a source data file.

As i) S100A9 suppresses diabetic ketogenesis and ii) ketone bodies have been shown to inhibit mTORC1 signaling in the kidney^51^, it is formally possible that the change induced by S100A9 on hepatic mTORC1 signaling is secondary to its ketone-lowering action. If this were to be the case, an increased hepatic mTORC1 signaling would be inconsequential to ketone body metabolism in diabetes. Thus, to directly address the role of hepatic mTORC1 in diabetic ketogenesis, we generated ID mice homozygous for the loxP-flanked *Tuberous Sclerosis Complex 1* (*Tsc1)* allele (a negative regulator of mTORC1 activity)^52^. To achieve liver-specific *Tsc1* deletion these mice were infected with either Adenovirus-Cre-GFP (referred to as *Tsc1*^*liver-KO*^ mice) or Adenovirus-GFP (referred to as *Tsc1*^*fl/fl*^ mice) as controls and both groups were rendered insulin deficient (Fig. 3A, B) (see Methods for details). ID-*Tsc1*^*liver-KO*^ mice show reduced hepatic *Tsc1* mRNA level compared to ID controls; yet, it remains unchanged in skeletal muscle (Fig. 3C). Moreover, in accordance with the fact that TSC1 is an mTORC1 inhibitor, they have increased hepatic pS6/S6 content compared to their TSC1-intact ID controls (Fig. 3D). Remarkably, despite the fact that both genotypes had a similar degree of severe ID (Fig. 3B) the ID controls developed overt hyperketonemia; yet, *Tsc1*^*liver-KO*^ ID mice exhibited a significant reduction in circulating and hepatic β-hydroxybutyrate level with latter being normalized (Fig. 3E, F). Like our previous findings shown in Supplementary Fig. 1I, J and Supplementary Fig. 2S we could not detect a change in either circulating NEFAs level or adipose tissue lipase activity between *Tsc1*^*liver-KO*^ ID mice and their *Tsc1*^*fl/fl*^ ID controls (Fig. 3G, H) indicating a liver specific effect on ketone body production. Collectively, these data reveal hepatic mTORC1 as a downstream intracellular molecular component of the S100A9-TLR4 pathway able to suppress diabetic ketogenesis.

**Fig. 4:**
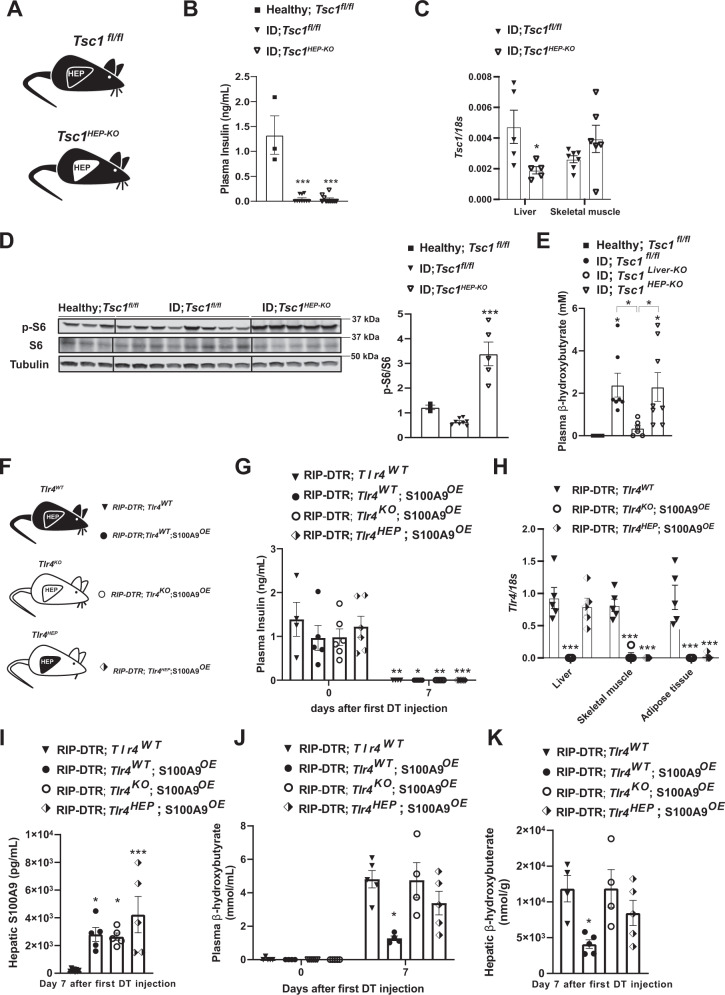
S100A9 suppresses diabetic ketogenesis by activating hepatic mTORC1/TLR4 axis in non-parenchymal hepatic cells. **A** Experimental groups. Homozygous Tuberous sclerosis complex 1 floxed (*Tsc1*^*fl/fl*^) mice were administered 1 × 10^9 PFU Adenovirus serotype 8 expressing Cre and GFP (Cre recombinantion results in a *Tsc1* null allele in the hepatocytes of *Tsc1*^*fl/fl*^ mice): *Tsc1*^HEP-KO^. Control mice were treated with 1 × 10^9 PFU Adenovirus serotype 8 expressing only GFP: *Tsc1*^*fl/fl*^ and analysed alongside healthy controls (*n*/group = 3, 8 and 5). **B** Plasma insulin levels of insulin deficient mice and healthy controls (*p* = 0.0001). **C** mRNA content of *Tcs1* in the liver (*p* = 0.0571) and skeletal muscle (gastrocnemius) of the indicated groups. **D** Image of immunoblot for the indicated proteins and phosphorylation states and relative densitometry quantification of pS6/S6 (*p* = 0.006). **E** Plasmatic β-hydroxybutyrate levels of indicated groups (*p* = 0.028 and *p* = 0.055). **F** Experimental groups (*n*/group = 4, 5, 6 and 5). **G** Plasma insulin levels of mice at day 0 (i.e., before DT injection) and 7 days after first DT injection (*p* = 0.001, *p* = 0.022, *p* = 0.0076 and *p* = 0.0005). **H** mRNA content of *Tlr4* in the liver, muscle (gastrocnemius), and adipose tissue (interscapular brown adipose tissue) of indicated groups (*p* = 0.0001). **I** Hepatic S100A9 content of indicated cohorts 7 days after first DT injection (*p* = 0.017, *p* = 0.027 and *p* = 0.0004). **J** Plasmatic hepatic β-hydroxybutyrate levels (*p* = 0.013). **K** hepatic β-hydroxybutyrate levels in the indicated cohorts and time after first DT injection (*p* = 0.021). Error bars represent SEM, statistical analyses were done using one-way or two-way ANOVA (Tukey’s post- hoc test). Comparisons were made to RIPDTR; *Tlr4*^WT^ group or in **B** and **D** to to healthy group or otherwise indicated. *p ≤ 0.05, **p ≤ 0.01, ***p ≤ 0.001. Source data are provided as a source data file.

To directly investigate the role of increased mTORC1 in parenchymal hepatic cells (i.e., hepatocytes), we performed similar experiments by using an adenovirus expressing Cre-recombinase under the control of the thyroid hormone binding globulin (TBG) promoter (Adenovirus-TBG-Cre/GFP), and hence delivering Cre-recombinase solely in hepatocytes^53^. We generated *Tsc1*^*fl/fl*^ ID mice and infected them with either Adenovirus-TBG-Cre (referred to as *Tsc1*^*HEP-KO*^ mice) or Adenovirus-TBG-GFP (referred to as *Tsc1*^*fl/fl*^ mice) (Fig. 4A, B). Widespread expression of Adenovirus-TBG-GFP was observed in hepatocytes; yet its expression was undetectable in non-parenchymal hepatic cells as for example Kupffer cells (Supplementary Fig. 2T). ID *Tsc1*^*HEP-KO*^ mice show reduced hepatic *Tsc1* mRNA and increased pS6/S6 protein levels compared to ID controls (Fig. 4C, D). However, unlike ID-*Tsc1*^*liver-KO*^ mice, ID *Tsc1*^*HEP-KO*^ mice showed no reduction in plasma β-hydroxybutyrate levels compared to *Tsc1*^*fl/fl*^ ID controls (Fig. 4E). These results demonstrate that increased mTORC1 activity specifically in hepatocytes is not sufficient to suppress diabetic ketogenesis and therefore suggests that enhanced mTORC1 activity in non-parenchymal hepatic cells suppresses ID-induced ketogenesis.

The aforementioned unexpected results spurred another important question: is TLR4 in hepatocytes sufficient for mediating the effect of S100A9 on ID-induced ketogenesis? To address this question, we generated ID mice with or without S100A9 overexpression allowing to re-express TLR4 only in hepatocytes. To accomplish hepatocyte-specific TLR4 expression we infected mice homozygous for the *Tlr4*^*LoxTB*^ allele and carrying the *RIP-DTR* allele with Adenovirus-TBG-Cre or GFP control virus (Fig. 4F). Plasma insulin was equally undetectable in all groups of mice 7 days after the first DT injection (Fig. 4G). This was accompanied by a specific regain of *Tlr4* mRNA in the liver of the Adenovirus-TBG-Cre treated group, without leakage into other metabolic tissues (Fig. 4H). Once again, hepatic S100A9 content was significantly increased in all groups given HTVI of the S100A9 plasmid (Fig. 4I). However, hepatocyte specific regain of TLR4 did not lead to a reduction of neither plasma nor hepatic ketone levels (Fig. 4J, K). Collectively, with the data shown in Fig. 1, our data demonstrate that TLR4 in non-parenchymal hepatic cells mediate the ketone body normalizing effect of S100A9 in ID.

### Extracellular S100A9 activates mTORC1 signaling in a cell-autonomous fashion

**Fig. 5:**
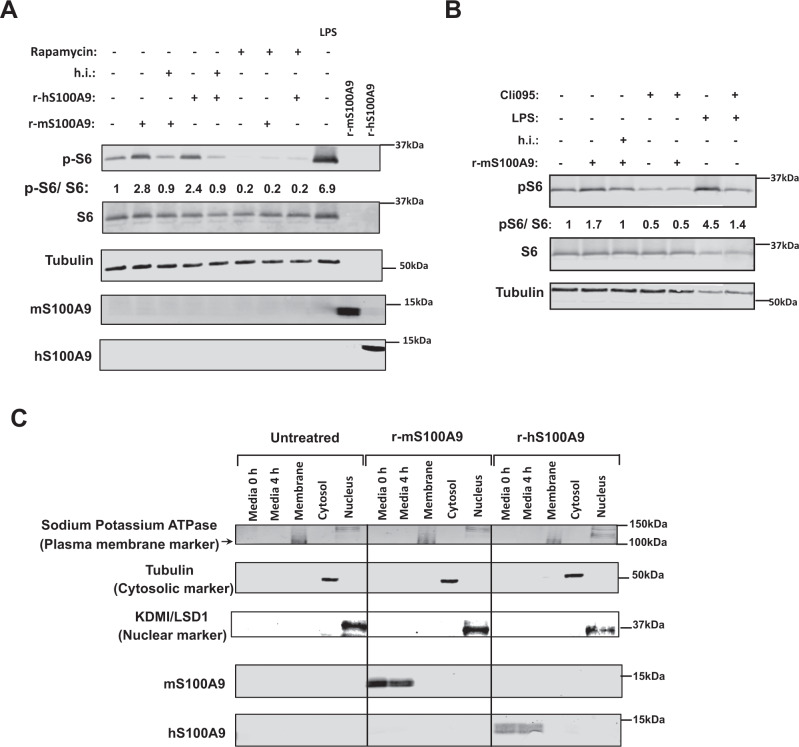
Extracellular S100A9 activates mTORC1 signaling in a cell-autonomous fashion. **A** RAW264.7 cells were incubated with 2 μg/mL of r-mS100A9 or r-hS100A9 (or their heat inactivated (h.i) forms) or 1 μg/mL of lipopolysaccharides (LPS, from *E.coli* O111:B4, Sigma) for 4 h, with or without 20 uM Rapamycin and cell lysates were analyzed by western blot. **B** RAW264.7 cells were incubated wit 2 μg/mL of r-mS100A9 or (or its heat inactivated form) in the presence or absence of 1 μg/mL of Cli095 (Invivogen) and cell lysates were analyzed by western blot. **C** RAW264.7 cells were incubated with 2 μg/mL of r-mS100A9 or r-hS100A9. Cells were fractionated and membrane, cytosolic and nuclear fractions were subjected to western blot alongside culture media. (*n* = 1 × 10^6 cells examined). Western blot images shown in **B** are cropped images. Source data are provided as a source data file.

Our results indicate that S100A9 activates mTORC1 signaling. Yet, S100A9 overexpression was achieved by HTVI; an approach leading to increased intracellular and extracellular S100A9 content^42^. Therefore, from a translational viewpoint it is very important to address whether the beneficial effects i) are induced by extracellular and/or intracellular S100A9 and ii) reproduced by S100A9-based therapeutic(s) (e.g., recombinant S100A9). To begin addressing these questions, we generated recombinant murine S100A9 (r-mS100A9) protein as described in the Methods section. r-mS100A9 shows the expected molecular weight and alpha-helical structures^54^ (Supplementary Fig. 3A, B). Addition of r-mS100A9 to the culture media increased pS6/S6 content in RAW264.7 cells (murine cells expressing TLR4) (Fig. 5A, B). This result was not due to the presence of endotoxin contaminants in the r-mS100A9 solution as heat-inactivated r-mS100A9 was unable to increase pS6/S6 level in these cells (Fig. 5A, B). Moreover, and further supporting the role of mTORC1 in mediating the S100A9-induced phosphorylation of S6, the ability of r-mS100A9 to increase pS6/S6 level was blunted by rapamycin (an mTORC1 inhibitor) (Fig. 5A). These results were additionally confirmed by using a fluorescent reporter of mTORC1 signaling (mCherry-TOSI)^55^. This system is based on the expression of a fusion protein of programmed cell death 4 (PDCD4) and mCherry. Once mTORC1 is activated, PDCD4 is rapidly phosphorylated by S6 kinase (Supplementary Fig. 6K) and ubiquitinated, leading to proteasomal degradation. Therefore, abundance of mCherry is inversely proportional to mTORC1 activity. Indeed, our data show that the mCherry fluorescence content decreases after treatment with r-mS100A9 (indicating increased mTORC1 activity) and that this effect is blunted by exposure to rapamycin (Supplementary Fig. 3C). Additionally, and in accord to our in vivo data (Fig. 2C, D), the mTORC1 activation by r-mS100A9 was dependent on TLR4 signaling as treatment with CLI-095 (a specific inhibitor of TLR4 signaling) prevented r-mS100A9-mediated increase of pS6/S6 level (Fig. 5B). We also produced recombinant human S100A9 (r-hS100A9). This protein shows the expected molecular weight and alpha-helical structures^54^ (Supplementary Fig. 3A, B). It also shows a similar oligomerization status as r-mS100A9 (Supplementary Fig. 3D), expectedly a homodimeric status (Supplementary Fig. 3E) which we confirmed by size-exclusion-chromatography multi-angle light scattering analysis (Supplementary Fig. 3F). Of note, despite only 60% sequence homology between human and murine S100A9 (Supplementary Fig. 3G), r-hS100A9 protein exerted similar action as r-mS100A9’s. Indeed, addition of r-hS100A9 to the culture media increased pS6/S6 content in RAW264.7 cells; an effect that was blunted by heat exposure or rapamycin (Fig. 5A). To rule out the possibility that the aforementioned effect is idiosyncratic to the murine RAW264.7 cell type, we performed similar experiments in human THP1 cells. Analogous to the outcome obtained with murine cells, treatment with r-mS100A9 or r-hS100A9 increased pS6/S6 content in human THP1 cells and this effect was blunted by heat exposure or rapamycin (Supplementary Fig. 3H).

By taking advantage of the poor sequence homology between human and murine S100A9 (Supplementary Fig. 3C), we directly assessed whether r-hS100A9 added to the culture media is internalized in murine cells. First, the antiserum against human or murine S100A9 does not recognize murine or human S100A9, respectively. Indeed, while the antiserum against human S100A9 detected S100A9 in the culture media of RAW264.7 cells treated with r-hS100A9 it failed to do so in the culture media of cells treated with r-mS100A9 (Fig. 5C). In addition, while the antiserum against mouse S100A9 detected S100A9 in the culture media of RAW264.7 cells treated with r-mS100A9 it failed to do so in the culture media of cells treated with r-hS100A9 (Fig. 5C). Hence, consistent with the idea that r-hS100A9 is not internalized and acts extracellularly, the antiserum against human S100A9 was not able to detect S100A9 in the membrane, cytosolic, or nuclear fractions of RAW264.7 murine cells; yet, it promptly detected S100A9 in the culture media of these cells treated with r-hS100A9 (Fig. 5C). Of note, RAW264.7 cells do not express detectable level of endogenous S100A9 as indicated by the fact that the antiserum against murine S100A9 was not able to reveal S100A9 in the membrane, cytosolic, or nuclear fractions of these cells; however, it detected S100A9 in the culture media of these cells treated with r-mS100A9 (Fig. 5C). Collectively, these data reveal that activation of mTORC1 signaling by S100A9 does not require internalization of S100A9 into the cells and therefore S100A9 acts via an extracellular modality.

### Efficacy and safety of recombinant S100A9 administration in vivo

**Fig. 6:**
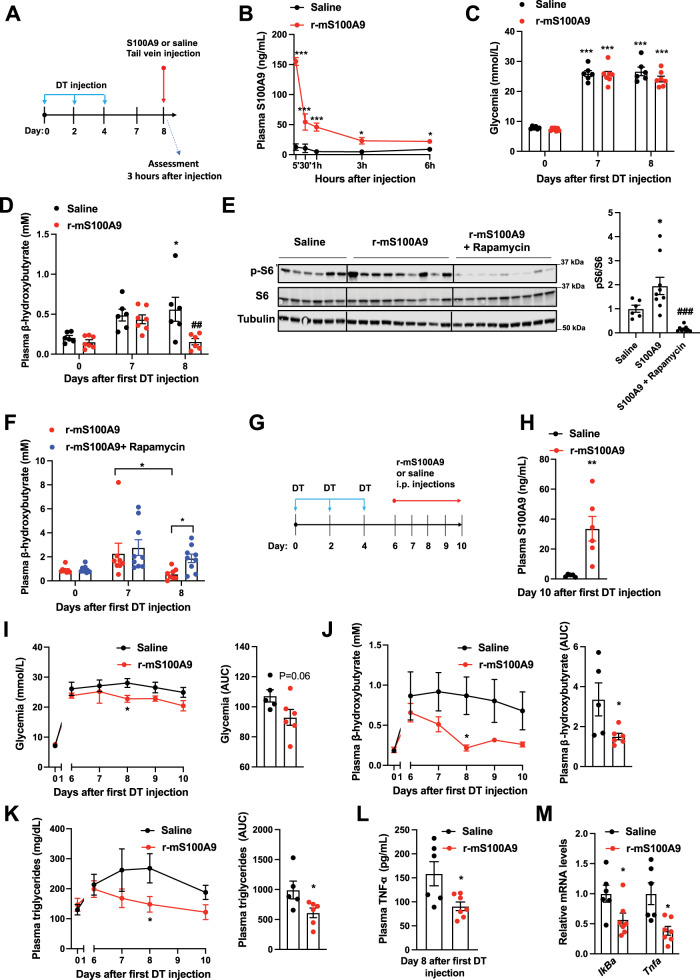
Efficacy and safety of recombinant S100A9 administration in ID mice. **A** RIP- DTR mice were treated at day 0, 2, and 4 with DT and at day 8, were injected via tail vein with either saline (*n* = 6) or 0.6 mg/kg of r-mS100A9 (*n* = 7) (alone or in combination with intraperitoneal injection with 10 mg/kg of rapamycin) (*n*/group = 6, 9 and 9). Metabolic assessments were done 3 h after injection and food removal. **B** Plasma level of S100A9 after injection of r-mS100A9 or saline (*p* = 0.001, *p* = 0.001, *p* = 0.001, *p* = 0.02, *p* = 0.03). **C** Glycemia (*p* = 0.001) and **D** plasma level of β-hydroxybutyrate of ID mice treated with 1 injection of r-mS100A9 or saline (*p* = 0.04 and *p* = 0.007). **E** Immunoblot from liver lysates and on the right bars indicating relative quantification of pS6/S6 (*p* = 0.012 and *p* = 0.0002). **F** Plasma level of β-hydroxybutyrate of ID mice treated with 1 injection of r-mS100A9 alone or in combination with rapamycin (rapamycin was injected at the same time of r-mS100A9 and values taken 3 h later) (*p* = 0.016 and *p* = 0.055). **G** RIP-DTR mice were treated at day 0, 2, and 4 with DT and starting from day 6, were intraperitoneally injected 2 times/day (9 am and 6 pm) with either 0.6 mg/kg of r-mS100A9 (*n* = 6) or saline (*n* = 5) (200 μl total volume) and here we show their values of **H** plasma S100A9 (*p* = 0.008), **I** daily glycemia (*p* = 0.01) (and area and the curve of glycemia from day 6), and **J** daily ketonemia (*p* = 0.015 and *p* = 0.038) (and area and the curve of ketonemia from day 6), **K** Daily triglyceridemia (*p* = 0.048) and area under the curve of tryglyceridemia from day 6) (*p* = 0.042). **L** Plasma level of TNF-a, (*p* = 0.02) and **M** level of hepatic *IkBa* (*p* = 0.036) and *Tnfa* (*p* = 0.017) mRNA. In all groups, glycemia and plasma were obtained at noon after 3 h of food removal. Error bars represent SEM. In **B**–**F** and **I**–**K**, statistical analyses were done using one or two-ways ANOVA (Tukey’s post-hoc test) except in 6 F where FDR was used * and **#** indicate comparison to basal and to saline treatment (at the same time), respectively. Statistical analyses in **H**, **L** and **M** were done using a two-tailed unpaired Student’s t-test. *P < 0.05, **P < 0.01, ***P < 0.001, ^#^*P* < 0.05, ^##^*P* < 0.01, ^###^*P* < 0.001. Source data are provided as a source data file.

Our data indicate that S100A9 acts in an extracellular manner; hence means aimed at increasing plasmatic S100A9 content could be of therapeutic value in context of diabetes. Thus, we directly tested whether in vivo treatment with recombinant S100A9 is effective and safe. First, we performed tail vein delivery of r-mS100A9 in DT-treated *RIP-DTR* ID mice and found that this approach leads to a rapid increase in plasmatic S100A9 level that remained significantly higher (compared to saline-injected controls) for at least 6 h after injection (Fig. 6A, B and Supplementary Fig. 4A). Remarkably, r-mS100A9 administration rapidly normalized hyperketonemia and slightly improved hyperglycemia in these ID mice (Fig. 6C, D). Mechanistically, and in line with our data shown in Fig. 2, the hyperketonemia-lowering action of r-mS100A9 requires mTORC1 as the ability of r-mS100A9 to i) increase hepatic pS6/S6 level and ii) normalize diabetic hyperketonemia is significantly blunted by rapamycin (Fig. 6E, F). We also found that rapamycin treatment alone was not able to induce a further increase in circulating ketone bodies (Supplementary Fig. 4B). Importantly, the ability of S100A9 to reduce expression of PPARα’s target genes (Fig. 2E) was suppressed by rapamycin treatment (Supplementary Fig. 4C).

Diabetes is a chronic disease; therefore, we also assessed the outcome of prolonged r-mS100A9 administration. DT-treated *RIP-DTR* mice developed ID, hyperglycemia, hyperketonemia, and hypertriglyceridemia and underwent twice-a-day intraperitoneal injection of either r-mS100A9 or saline, for 4 days (Fig. 6G–K and Supplementary Fig. 4D). This protocol led to increased circulating level of S100A9 in r-mS100A9- compared to saline-injected ID mice (Fig. 6H). During the treatment period, r-mS100A9-injected ID mice displayed a slight amelioration in hyperglycemia and a marked improvement in hyperketonemia and hypertriglyceridemia compared to their controls (Fig. 6I–K). Plasma NEFAs level and adipose tissue lipase activity were not changed in the S100A9 treated groups (Supplementary Fig. 4E, F). Collectively, these results indicate that the metabolic-improving effect of recombinant S100A9 therapy is maintained during chronic treatment.

While the aforementioned data support the idea that S100A9-based therapy is effective, for a translational application a new drug must also be safe. Thus, we assessed the safety profile of recombinant S100A9 therapy in ID mice. Considering that TLR4 i) mediates the beneficial effects of S100A9 and ii) is involved in the inflammatory response, a potential important safety concern of S100A9-based therapy is increased inflammation^36^. To directly address this issue, we measured several parameters that are induced upon inflammation. Surprisingly, we found that S100A9 treatment exerts an anti-inflammatory effect on ID mice. In fact, the level of the circulating inflammatory cytokine TNF-α were significantly reduced by r-mS100A9 treatment (Fig. 6L). TLR4 activation leads to increased NF-kB signaling^56^. Yet, consistent with an anti-inflammatory effect, we found that hepatic mRNA contents of NF-kB target genes such as *IkBa* and *Tnfa* were also reduced by r-mS100A9 treatment (Fig. 6M). Additionally, expected signs of deleterious TLR4 activation (e.g. the ones caused by lipopolysaccharides treatment) such as piloerection, lethargy, diminished or irregular respiration^57^ were never observed after administration of r-mS100A9. Moreover, as deleterious TLR4 signaling impacts energy balance, we assessed food intake, body weight, fat, and lean mass and found no differences in these parameters between r-mS100A9- and saline-treated ID mice (Supplementary Fig. 4G–J). Thus, these data suggest that r-mS100A9 treatment does not bring about adverse effects, and in the context of insulin deficiency can even promote an anti-inflammatory action (an effect which could potentially bring additional therapeutic benefits). Hence our proof-of-concept results support the notion that recombinant S100A9 is an effective and safe anti-diabetic therapeutic.

**Fig. 7:**
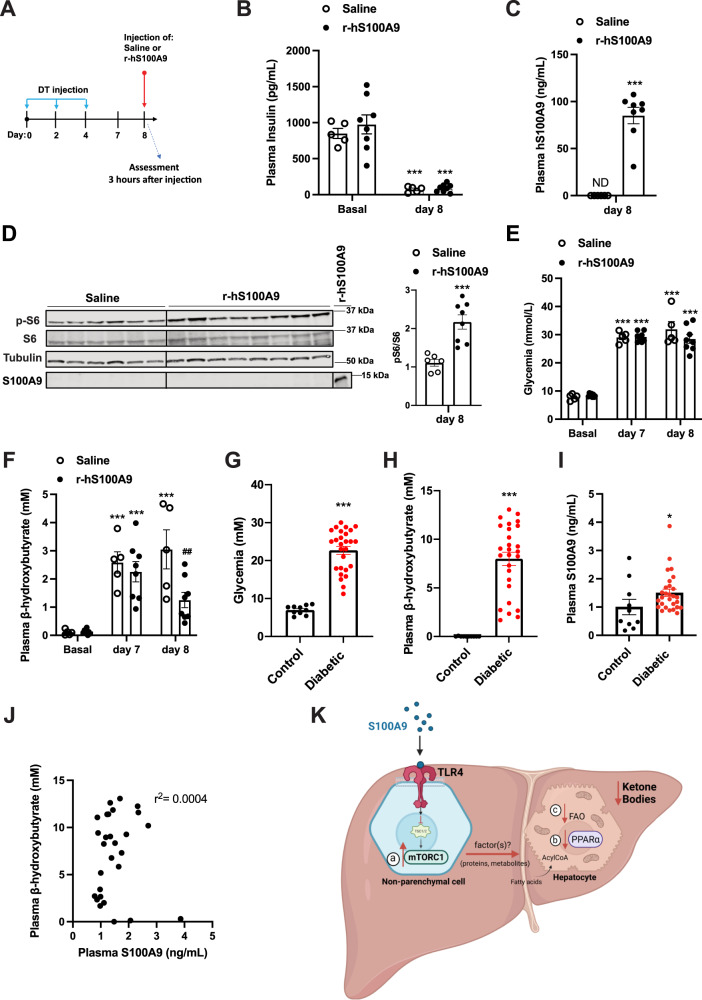
Clinical relevance of recombinant S100A9 as an anti-diabetic therapeutic. **A** RIP- DTR mice were treated at day 0, 2, and 4 with DT and at day 8, were injected via tail vein with either saline (*n* = 5) or 0.6 mg/kg of r-hS100A9 (*n* = 8). Metabolic assessments were done 3 h after injection and food removal. **B** Plasma insulin level (*p* = 0.0001), **C** human S100A9 plasma level (*p* = 0.0001), **D** indicated hepatic protein levels (*p* = 0.0006), **E** glycemia (*p* = 0.0001), and **F** ketonemia (*p* = 0.001, *p* = 0.001, *p* = 0.001 and *p* = 0.009), of ID mice treated with 1 injection of saline or r- hS100A9. **G** Glycemia (*p* = 0.0001). **H** Ketonemia (*p* = 0.0001), and **I** plasmatic S100A9 content in healthy (*n* = 10) and decompensated diabetic subjects (*n* = 23) (*p* = 0.034). **J** Correlation analysis between ketone and S100A9 levels in decompensated type 1 diabetic subjects. **K** Our model predicts that extracellular S100A9 activates non-parenchymal hepatic TLR4 signaling which consequently leads to several downstream events. These include a) activation of the mTORC1 pathway, b) dampening of the PPARα targets and c) reduction of fatty acid oxidation in hepatocytes all of which contribute to the significant regression of the elevated ketogenesis caused by pancreatic beta-cell loss/dysfunction. This figure was created with BioRender.com. Error bars represent SEM. Statistical analyses in **C**, **D** and **G**–**I** were done using two-tailed unpaired Student’s *t*-test. Statistical analyses in **B**, **E** and **F** were done using two-ways ANOVA (Tukey’s post-hoc test). * and **#** indicate comparison to basal and to saline treatment (at the same time), respectively (except when differently indicated: in **F**). The correlation analysis in **J** was performed by using Spearman rank-correlation test. *P < 0.05, **P < 0.01, ***P < 0.001, ^##^*P* < 0.01. Western blot images shown in **D** are cropped images. Source data are provided as a source data file.

The recombinant human S100A9 (r-hS100A9) is the suitable clinical form of S100A9. Of note, our data show that despite the poor homology in amino acids sequence between human and murine S100A9 (Supplementary Fig. 3C), r-hS100A9 activates mTORC1 signaling in a murine cell line (Fig. 4A). These data suggest the possibility that r-hS100A9 can also be effective in vivo in ID mice. To directly test this possibility, we performed tail vein delivery of either r-hS100A9 or saline in DT-treated *RIP-DTR* mice (Fig. 7A, B). Three hours after injection, r-hS100A9-injected ID mice showed a plasmatic concentration of hS100A9 similar to what was  achieved in r-mS100A9-injected ID mice (Figs. 6B, 7C). According to its effect on murine cells, r-hS100A9 treatment caused activation of hepatic mTORC1 signaling in ID mice as determined by increased hepatic pS6/S6 ratio compared to saline-treated controls (Fig. 7D). Importantly, in line with our in-vitro studies hS100A9 was not detected in the lysates of perfused liver of mice treated with r-hS100A9, further supporting the notion of an extracellular action of S100A9 (Fig. 7D). Remarkably, r-hS100A9 administration rapidly lowered hyperketonemia and there was also a trend towards improved hyperglycemia in ID mice (Fig. 7E, F). Moreover, supporting feasibility of r-hS100A9 treatment, we also found that after subcutaneous injection (a suitable route of administration for chronic treatment in human) r-hS100A9 was greatly bioavailable with a plasma pharmacokinetic that was similar to the one obtained with tail vein injection (Supplementary Fig. 4K).

To further evaluate the translational significance of our findings, we performed an observational clinical study aimed at gathering plasmatic S100A9 content in patients with decompensated diabetes and healthy controls. As expected, all patients were hyperglycemic and hyperketonemic (Fig. 7G, H). In this cohort, we observed a modest increase of circulating S100A9 content compared to healthy subjects (Fig. 7I). However, this change is insufficient to produce any hyperketonemia-lowering effect as no correlation was observed between plasmatic level of S100A9 and ketones (Fig. 7J). We also found no correlation in circulating S100A9 and HbA1c or glycemia and no significant differences in control and patient S100A9 levels based on age or gender (Supplementary Fig. 4L–O). Of note, the slight increase in plasmatic S100A9 level observed in poorly controlled subjects with diabetes is also recapitulated in our ID mouse model^42^. Therefore, as we demonstrated that increasing the circulating level of S100A9 is able to improve the metabolic imbalance caused by ID (Fig. 6C, D, I–K), our murine and human data provide scope for further increasing plasmatic S100A9 as a potential therapeutic clinical avenue in diabetes.

## Discussion

Our results uncover the hepatic S100A9-TLR4-mTORC1 axis as an insulin independent mechanism capable of suppressing diabetic hyperketonemia. This conclusion is supported by in vivo data gathered from murine models of insulin deficiency (ID) i) lacking or re-expressing TLR4 only in the liver, ii) displaying increased mTORC1 activity only in the liver, and iii) treated with recombinant murine S100A9 with or without rapamycin (an mTORC1 inhibitor) and their controls. Supporting the translational significance of our findings, we also report that similarly to its murine ortholog recombinant human S100A9 administration is remarkably effective in reducing hyperketonemia of ID mice. Coupled with the only marginal increase in plasmatic S100A9 in decompensated patients with diabetes, our data are in favor of the development of an S100A9-based adjunctive therapeutic approach aimed at achieving better metabolic control in patients with diabetes suffering from ID. As indicated in Fig. 7K and based on our results, we propose a model in which administration of human recombinant S100A9 acts on TLR4 in the liver to trigger the mTORC1 axis which, in part via suppression of PPARα, leads to reduced hepatic fatty acid oxidation (FAO) and ketogenesis in an insulin-independent fashion in patients suffering from ID.

Based on our data we suggest that TLR4 and mTORC1 in non-parenchymal hepatic cells mediate the effects of S100A9 on ID-induced ketogenesis (Fig. 7K). Noteworthy, previous studies directly tested whether enhanced mTORC1 activity in the liver (i.e., in parenchymal and non-parenchymal cells)^10,16^ or specifically in hepatocytes^58^ is sufficient for suppressing ketogenesis in a physiological context (i.e. following prolonged fasting). Interestingly, while enhanced mTORC1 activity in the liver is able to suppress fasting-induced ketogenesis its overactivity only in hepatocytes failed to achieve this effect^10,16,58^. Our assessment of the results from the aforementioned three studies in combination with our data shown herein would shift the focus from the ketogenic-suppressing role of mTORC1 in hepatocytes (as previously thought and debated)^58^ to mTORC1 in non-parenchymal hepatic cells. Although the precise identity of which non-parenchymal hepatic cell type(s) mTORC1 (and TLR4) are mediating changes in hepatocyte ketogenesis remain to be elucidated our results will spur new studies aimed at identifying in which and how these yet-to-be-identified mTORC1-expressing cells communicate to hepatocytes to regulate ketogenesis in health (e.g., following prolonged fasting) and disease (e.g., ID). Therefore, further investigations aimed at manipulating TLR4 and/or mTORC1 activity exclusively in each of the non-parenchymal hepatic cell-types (e.g., cholangiocytes, stellate, Kupffer, endothelial cells and others) are warranted. Additionally, due to estrous cycle mediated metabolic variations in females that could present as confounding factors, only male mice have been used for this study. Future studies aimed at assessing the generalizability of these results to female mice are warranted.

In the context of ID hyperketonemia is supported by enhanced rate of adipose tissue lipolysis; an effect leading to increased availability of circulating NEFAs that are then used as substrate to generate acetyl-CoA from FAO in the liver^6^. Our data support a model (Fig. 7K) in which changes in hepatic FAO (and not in adipose tissue lipolysis) underlie the effect of S100A9 on ID-induced ketogenesis. In fact, our data indicate that S100A9 overexpression or treatment does not cause changes in circulating NEFA levels or adipose tissue lipolysis but can significantly suppress hepatic FAO (Fig. 2G, Supplementary Fig. 1I, J, Supplementary Figs. 2S, 3G, H and Supplementary Fig. 4E, F). These results are in line with our previously published data^42^. Furthermore, a direct effect of S100A9 on adipose tissue is ruled out by the data shown in Fig. 1E, F demonstrating that ID mice expressing TLR4 only in the liver (and therefore unable to express TLR4 in adipose tissue and all other tissues) responded properly to the hyperketonemia-lowering action of S100A9. If TLR4 in adipose tissue were to be required for this effect of S100A9 the aforementioned animal model should have been unable to respond to the hyperketonemia-lowering action of S100A9, which was not the case.

Despite continuing advances in ID therapy, current methodologies are still sub-optimal in achieving adequate metabolic control in patients with diabetes^59^. This shortcoming might be due to the fact that the current approach to diabetes treatment is chiefly glucose-centric, with virtually all available, and investigational, adjunctive therapies aiming at improving hyperglycemia^60^. However, in addition to hyperglycemia ID is accompanied by other deleterious metabolic consequences (e.g., hyperketonemia, hyperglucagonemia, and hypertriglyceridemia) that have received comparatively little attention. Indeed, an increasing body of evidence is emerging that challenges the prevailing idea that reducing hyperglycemia is a unifying target in diabetes treatment, particularly for the prevention of long-term complications. The development of diabetic neuropathy, for instance, has been shown to be independent of normalizing hyperglycemia^61^. Our previous study also indicates that a significant extension of lifespan of mice with pancreatic β-cell loss and ID could be achieved without dramatic improvements in hyperglycemia^42^. Furthermore, many clinical studies highlight that the prevalence of long-term complications in diabetes are independent of glycemic control^62–64^. This underscores the need to extend treatment approaches beyond correcting hyperglycemia. Diabetic ketoacidosis (DKA), for example, is a life-threatening complication whose incidence remains high in people suffering from diabetes^19^. Additionally, and in line with the glucose-centric view of diabetes, treatment with adjunctive therapies based on SGLT1/2 inhibitors have been reported to decrease circulating glucose level; yet, at the expenses of increase DKA risk^65^. Thus, there is a lack of pharmacological interventions that tackle this outstanding problem in patients with diabetes^65^. We envisage that our study provides the supporting data for further development of S100A9-based therapeutics. For example, based on our efficacy and pharmacokinetics data shown in Fig. 7A–F and Supplementary Fig. 4K and interspecies allometric scaling^66^ the expected human dose is less than 0.1 mg/kg; a dose anticipated to be feasible for pharmacotherapy. Also, an insulin/S100A9 pharmacotherapy approach owns the ability to concurrently activate parallel insulin-dependent and insulin-independent pathways. Notably, the loss of S100A9 itself does not worsen hyperketonemia in ID (Supplementary Fig. 1C). This, coupled with the lack of correlation between plasma S100A9 levels and β- hydroxybutyrate in human patients (Fig. 7J) suggests that pathophysiological S100A9 level is not sufficient to suppress ketone production; hence, supraphysiological level or S100A9 is required for its beneficial effects in ID. Importantly, our data also underscore the safety of a S100A9-based treatment. In particular, S100A9 acts via TLR4, yet it has anti-inflammatory action (Fig. 6L, M). Furthermore, S100A9 leads to only a mild physiological activation of mTORC1 activity (Fig. 2A), hence excluding concern regarding possible deleterious effects due to overactivation of this signaling (e.g., pro-oncogenic effects).

Therefore, this methodology holds the potential to reduce insulin needs and consequentially diminish the risk of its unwanted side effects (e.g., life-threatening hypoglycemia) while improving metabolic control. Thus, S100A9 is a realistic next-generation biological agent for treatment of ID, a disorder that is at present pandemic.

## Methods

### Animal studies

All animal research carried out in this study complies with all relevant ethical regulations of the University of Geneva, within the procedures approved by animal care and experimentation authorities of the Canton of Geneva, Switzerland (animal protocol numbers GE/78/18, GE/207/19). Mice were maintained with standard chow diet and water available *ad libidum* in a light and temperature-controlled environment with a 12 h light/dark cycle. All the experiments described in the study used male mice with mixed genetic background aged 2-3 months. Two insulin deficient animal models were generated and studied. To induce insulin deficiency, *RIP-DTR* mice previously studied^42^, weighing 25–30 g were used. Diphteria Toxin (DT, Sigma Aldrich) was dissolved in sterile 0.9% NaCl and i.p administrated and injected intraperitoneally as previously shown^42^. *Tsc1*^fl/fl^ and *Tsc1*^liver-KO^ mice were rendered insulin deficient by i.p injection of 150 mg/kg streptozotocin (STZ; Sigma Aldrich, dissolved in in sterile 0.9% NaCl), 4 days after adenoviral treatment. Metabolic assessments were performed 5 days after STZ injection. The *Tlr4*^*LoxTB*^ allele was generated as previously described^44^. F1 pups containing the *Tlr4*^*LoxTB*^ allele were bred with *RIP-DTR* mice to generate male and female *Tlr4*^*LoxTB/+*^; *RIP-DTR* mice, which were then bred together to generate *Tlr4*^*LoxTB/LoxTB*^; *RIP-DTR* mice and *Tlr4*^*+/+*^; *RIP-DTR* controls. Recombinant adenoviruses serotype 5 or serotype expressing either Cre recombinase and GFP concomitantly (Ad-Cre-GFP) or only GFP (Ad-GFP) were generated by VectorBiolabs (USA). 10-week-old mice were injected with 10^9^ PFU into tail vein in 200 µL of saline. The treatment was performed 3 days or 7 days after DT or STZ treatment, respectively. The overexpression of S100A9 using hydrodynamic tail vein injection in mice was performed according to previous studies^42^. In brief, overexpression of S100A9 is achieved by using pLIVE vectors (Myrus) that allow expression under the control of the albumin promoter. Each mouse received 50 μg of vector encoding for S100A9 or empty vector as control. Care of mice at University of Geneva was within the procedures approved by animal care and experimentation authorities of the Canton of Geneva, Switzerland. For acute treatment of mouse or human r-S100A9, 25 g *RIP-DTR* ID mice were fasted for 3 h and injected with either 15 µg of r-S100A9 dissolved in 200 µl saline, or saline alone, via the tail vain. Blood samples were collected after 3 h. For experiments involving rapamycin, mice were administered 10 mg/kg rapamycin in 200 ul saline via i.p injection. For chronic r-S100A9 treatment ID mice were administered a twice daily i.p injection of 0.6 mg/kg r-S100A9 or saline for 5 consecutive days. On the 5^th^ day mice were fasted for 3 h and blood and tissues collected for analysis. Care of mice at The number of mice used in each experiment is mentioned in the corresponding figure legend.

### Assessment of mRNA and protein content

Mice were sacrificed, tissues quickly removed, snap-frozen in liquid nitrogen and subsequently stored at –80 °C. RNAs were extracted using Trizol reagent (Invitrogen). Complementary DNA was generated by Superscript II (Invitrogen) and used with SYBR Green PCR master mix (Applied Biosystem, Foster City, CA, USA) for quantitative real time PCR (q-RTPCR) analysis. mRNA contents were normalized to *18* *s* mRNA levels. All assays were performed using an Applied Biosystems QuantStudio® 5 Real-Time PCR System. For each mRNA assessment, q-RTPCR analyses were repeated at least 3 times. Proteins were extracted by homogenizing samples in lysis buffer (Tris 20 mM, EDTA 5 mM, NP40 1% (v/v), protease inhibitors (P2714-1BTL from Sigma, St. Louis, MO, USA), then resolved by SDS-PAGE and finally transferred to a nitrocellulose membrane by electroblotting. Insulin-induced phosphorylation of proteins and basal proteins levels were assessed by using commercially available antisera as previously described^42^.

### Blood chemistry

Fed hormones/metabolites levels were determined by collecting tail blood from mice that were without food for 3 h. Fasted hormones/metabolites levels were assessed in mice provided only with water ad libitum and without food for the indicated period. Time at day at which blood was collected was the same between groups. Tail vein blood was assayed for glucose levels using a standard glucometer (Nova Biomedical). Plasma was collected by centrifugation (1000 xg) in EDTA-coated tubes (Kent Scientific) and assayed using the indicated commercially available kits: β-hydroxybutyrate (Sigma), insulin (Crystal Chem. Inc.), TNF-α (Biovision), IL1-β (Biovision) and IL-6 (Biovision), S100A9 (R&D) and NEFA (Wako Chemicals).

### Assessment of hepatic β-hydroxybutyrate and S100A9 content

10 mg of liver tissue was homogenized in lysis buffer (Tris 20 mM, EDTA 5 mM, NP40 1% (v/v), protease inhibitors (P2714-1BTL from Sigma, St. Louis, MO, USA) and centrifuged at 17950 x g for 15 min at 4 °C. Supernatant was then transferred into fresh Eppendorf tubes. For S100A9 analysis, lysates were further diluted 1:50 in lysis buffer (Tris 20 mM, EDTA 5 mM, NP40 1% (v/v), protease inhibitors (P2714-1BTL from Sigma, St. Louis, MO, USA) and analyzed for S100A9 content with ELISA (R&D). For hepatic beta-hydroxybutyrate, lysates were deproteinized with Perchloric acid and assayed using commercially available beta-hydroxybutyrate kit (Sigma) according to the manufacturer’s instructions.

### Production of recombinant murine and human S100A9

The cDNAs encoding murine and human S100A9 proteins were cloned into a pET29+ vector including a N-terminal 10x histidine tag and a maltose-binding protein (MBP) tag followed by a tobacco etch virus (TEV) protease-cleavage site for removal of the tags. All proteins were expressed in *Escherichia coli* BL21 DE3 (Invitrogen) cells. Protein expression was induced at an A_600_ of 0.6 by addition of 1 mM isopropyl-β-D-thiogalactopyranoside (IPTG). Cells were harvested after 16 h incubation at 20 °C by centrifugation at 4000 xg. Bacterial cells were resuspended in 200 mM NaCl, 50 mM Tris-HCl (pH 8.0), 3% glycerol and 5 mM ß-mercaptoethanol and subsequently lysed using an Emulsiflex C3 system (Avestin). The fusion proteins were separately loaded onto a Ni-NTA HisTrap HP column (Cytiva) and an imidazole gradient elution with a final concentration of 0.5 M imidazole was performed. Cleavage of the MBP and polyhistidine tag was done by proteolytic digestion using tobacco etch virus (TEV) protease and dialysed for 16 h in 2 L buffer containing 150 mM NaCl, 20 mM Tris-HCl (pH 8.0) and 5 mM ß-mercaptoethanol. Removal of cleaved tags was performed through a reverse affinity purification using an MBPTrap HP column and TEV protease by using a GSTrap HP column (Cytiva). As a final purification step, the protein was injected onto a size-exclusion chromatography column (Superdex 200 GL Increase, Cytiva) equilibrated with isotonic PBS buffer (137 mM NaCl, 2.7 mM KCl, 8 mM Na_2_HPO_4_, and 2 mM KH_2_PO_4_, pH 7.4) for protein injection into mice. Where stated, heat inactivation (h.i) of r-mS100A9 and r-hS100A9 was performed at 95 °C for 15 min. Analytical gel filtration was performed using a Superose 6 Increase 3.2/300 column (Cytiva).

### UV circular dichroism spectroscopy

Far-Ultraviolet CD spectra were recorded on a Chirascan spectropolarimeter (Applied Photophysics) in nitrogen atmosphere at room temperature using a 0.1 cm path length quartz cell. Each spectrum was recorded between 260 and 195 nm. The data were collected at a rate of 1 nm/s with a wave­length step of 1 nm and a time constant of 0.5 s.

Proteins were dissolved in PBS buffer (137 mM NaCl, 2.7 mM KCl, 8 mM Na2HPO4, and 2 mM KH2PO4, pH 7.4) and a dilution series was performed to improve peak saturation at lower wavelengths.

### Analytical size-exclusion chromatography coupled to multi-angle light scattering (SEC-MALS)

30 µL of each protein sample (at a concentration of 5 mg/mL) were loaded onto a Superose 6 Increase 10/300 GL column (Cytiva). BSA measurements served as a quality control and runs were performed as initial and final run. The Agilent Infinity 1260 II HPLC system is coupled to a Wyatt miniDAWN TREOS 3 light scattering detector and a Wyatt Optilab T-rEX refractive index detector. System control and analysis were performed using the Wyatt Astra 7.3.1 software.

### Assessment of ex vivo FAO rate

The ex vivo radioactive fatty acid oxidation measurements were performed by using ^14^C-palmitic acid following an established protocol^67^. Before radioactive measurements, the lysis was done starting from 250 mg of liver in 0.5 ml of lysis buffer, and 50 μl of lysate in addition to 150 μl total FAO reaction buffer. The reaction is performed at 37 °C × 60 min.

### Cell lines and cellular fractionation experiments

RAW264.7 cells (TIB-71^TM^)were maintained in DMEM (4.5 g/L glucose, 110 mg/L pyruvate, 4mM L-glutamine, 100 U/mL Pen/Strep) supplemented with 10% fetal bovine serum (PanBiotech). THP-1 cells (TIB-202 ^TM^) were cultured according to guidelines specified by ATCC (https://www.atcc.org/), from which both cell lines were obtained. Experiments were performed in 6 well plates at 80% of cell confluency. Cells were treated with 2 μg/mL of r-mS100A9 or r-hS100A9 (or their heat inactivated forms) or 1 μg/mL of lipopolysaccharides (LPS, from *E. coli* O111:B4, Sigma) for 4 h, with or without 20 µM Rapamycin (Sigma) or 1 μg/mL of Cli095 (Invitrogen). Proteins were quickly extracted with lysis buffer. r-mS100A9 or r-hS100A9 were heat inactivated via incubation at 80 °C for 30 min. For fractionation experiments, membrane fractions were isolated using Mem-PER™ Plus Membrane Protein Extraction Kit (Thermo Fisher). Cytosolic and nuclear fractions were further isolated using NE-PER™ Nuclear and Cytoplasmic Extraction kit (Thermo Fisher) and subjected to western blot alongside culture media.

### Immunohistochemistry analyses

Immunohistochemistry for GFP was performed using paraffin embedded liver tissue sections. Briefly, 6 μm-thick sections were mounted on glass slides and treated for paraffin removal by serial passages (each of them 5′) in xylene and then PBS containing decreasing concentration of ethanol (100%, 70%, 50%, and 0%). Antigen retrieval was performed by boiling samples in Na-citrate buffer (pH = 6) for 30 min. Sections were washed in PBS 3 times for 10 min each and then blocked with 3% bovine serum albumin in PBT-Azide (2.5 mL of Triton X-100 in 1000 mL of PBS-Azide) for 30 min. The sections were then incubated with 1:1000 primary antisera (see Supplementary Table 1) with 3% bovine serum albumin in PBT-Azide overnight. The sections were then rinsed with PBS six times for 10 min each. Next, sections were incubated with 1:500 Alexa Fluor 488 anti-rabbit secondary antisera (Thermo Fisher) with 3% normal donkey serum in PBT for 2 h and subsequently rinsed in PBS three times for 10 min each. Sections were mounted with Fluroshield mouting medium with DAPI and visualized using Axioscan Z.1 fluorescent microscope. Quantification of %GFP positive cells was performed on 7 evenly spaced sections at 20x magnification.

### LPS treatment

Eight-week-old *Tlr4*^*LoxTB/LoxTB*^; *RIP-DTR* mice and *Tlr4*^*+/+*^; *RIP-DTR* controls were fasted for 3 h and treated were treated with 1 mg/kg body weight LPS or saline by intraperitoneal injection. Blood was collected 1.5 h after injection by tail bleeding. Plasma TNFα concentrations were determined by ELISA (Biovision).

### Plasmatic ketones and S100A9 content in healthy and decompensated diabetic subjects

We collected blood samples from 10 healthy donors (5 male and 5 female) aged between 35-65 years and 23 people with decompensated diabetes (9 male and 14 female) aged between 2-68 years (age bracket details are included in Supplementary Tables 2 and 3). Patients were admitted to the Geneva University Hospital (HUG) before treatment aimed at correcting their ketoacidosis was initiated. This clinical study was approved by Swissethics (BASEC ID: 2017-00470) and aimed at providing evidence for, or against, the therapeutic use of S100A9 in the context of hyperketonemia. Written informed consent was obtained from all adult patients. For pediatric patients informed consent was obtained from parents or legal guardians. In all cases no monetary compensation for participation was given.

### Adipose tissue lipase activity

Lipase activity rate was measured in the perigonadal white adipose tissue (pWAT) by using the lipase activity colorimetric kit assay II (Biovision). Briefly, 50 mg of pWAT was homogenized in 4 volumes of Assay Buffer and centrifuged (13,000 × g, 10 min) to remove insoluble material. Samples were then diluted in the Assay Buffer and lipase activity was measured at 412 nm, using a standard curve, a positive and a negative control provided by the kit assay.

### Assessment of energy homeostasis

Body fat and lean mass were determined using the Echo-MRI-700 system and caloric intake was measured using the TSE labmaster system available in the core facility of the Faculty of Medicine of the University of Geneva (http://www.unige.ch/medecine/en/recherche/corefacilities/).

### Statistical analysis

Data sets were analyzed for statistical significance using PRISM (GraphPad, version 9, San Diego, CA) for a two-tail unpaired Student’s t test when two groups were compared or one-or two-way ANOVA (Tukey’s post-hoc test or FDR) when more groups were compared.

### Materials availability

Further information and requests for resources and reagents should be directed to and will be fulfilled by the Lead Contact, Roberto Coppari (roberto.coppari@unige.ch).

### Statistics and reproducibility

In all experiments, western blot and immunohistochemical analysis were repeated at least twice with similar results.

### Reporting summary

Further information on research design is available in the Nature Research Reporting Summary linked to this article.

## Supplementary information

Supplementary Information

Reporting Summary

## Data Availability

The source data underlying all main and supplementary figures are provided as a Source Data file. Source data are provided with this paper.

## Source data

Source Data
