# Supplementary material for: Hepatic non-parenchymal S100A9-TLR4-mTORC1 axis normalizes diabetic ketogenesis
Source: Nat Commun. 2022 Jul 15;13:4107. doi: 10.1038/s41467-022-31803-5 (PMC9287425; doi:10.1038/s41467-022-31803-5)
Supplement: Supplementary file 1 — Supplementary Information [file 41467_2022_31803_MOESM1_ESM.pdf]

Supplemental Information

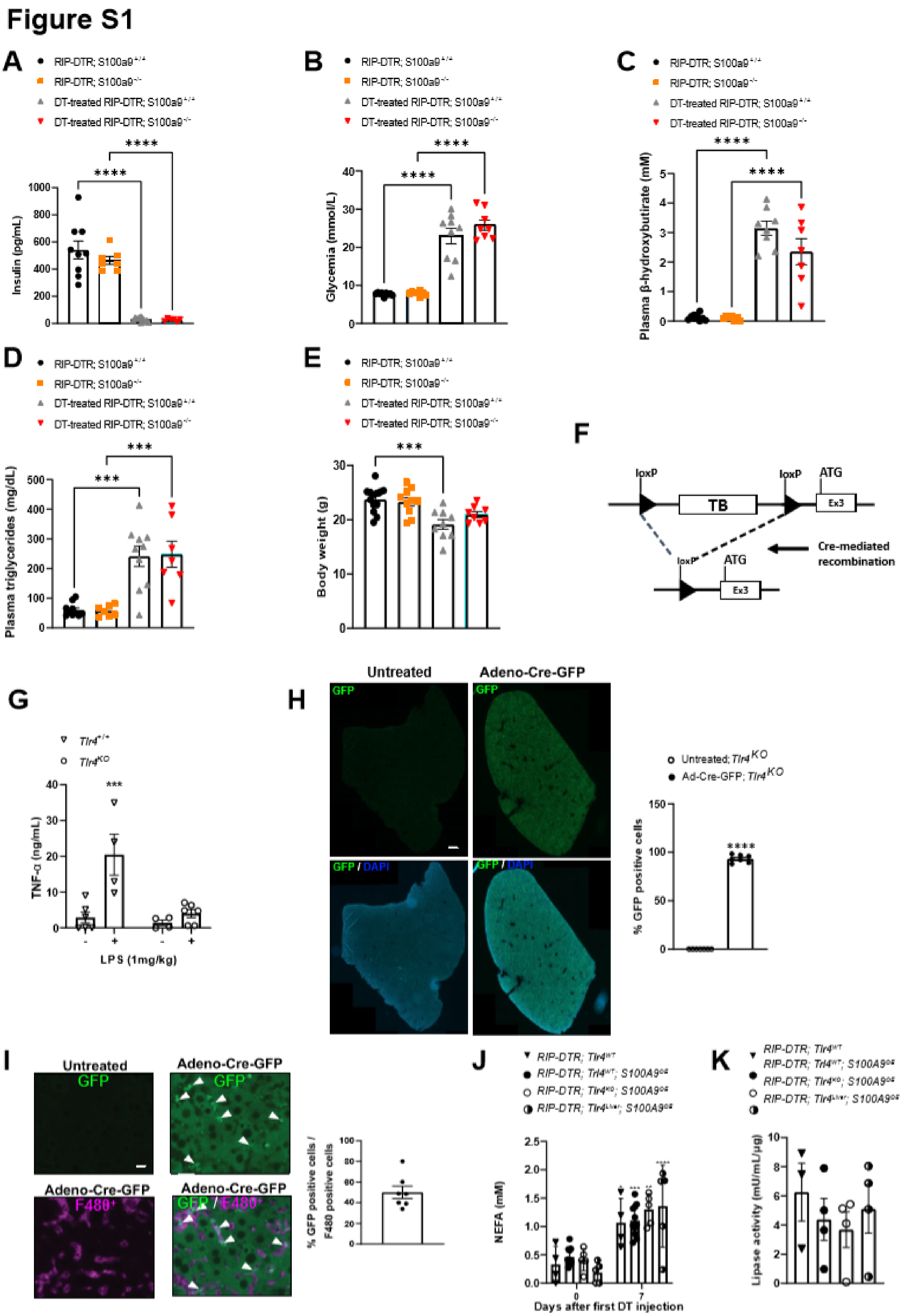

Supplemental figure 1. S100A9 suppresses diabetic ketogenesis via hepatic TLR4

(**A**) Plasma insulin (n/group = 7-9) (p=0.0001), (**B**) glycemia (n/group = 7-13) (p=0.0001), (**C**) plasma β-hydroxybutyrate levels (n/group = 7-10) (p=0.0001), (**D**) plasma triglycerides (TG) (n/group = 7-10) (p=0.0001, p=0.0006), and (**E**) Body weight (n/group = 7-13) of 12-week-old DT-untreated *RIP-DTR*; *S100a9*$^{-/-}$ and *RIP-DTR*; *S100a9*$^{+/+}$ mice and DT-treated *RIP-DTR*; *S100a9*$^{-/-}$ and *RIP-DTR*; *S100a9*$^{+/+}$ mice (p=0.0003) (pertinent to the DT-treated groups, samples were collected 7 days after the last DT injection). To avoid post-prandial confounding effects, data shown in **A-D** were collected from mice after 3 hours of fasting. (**F**) Schematic of the *Tlr4LoxTB* allele and PCR analysis of genomic DNA isolated from *Tlr4*^{WT} and *Tlr4*$^{LoxTB/+}$ mice (**G**) Lipopolysaccharide (LPS, 1mg/kg body weight) was injected intraperitoneally to chow-fed mice. Blood was collected 1.5 h after LPS administration and plasma TNF-α levels were measured by Elabscience ELISA (n/group = 4–6) (p=0.001). (**H**) Representative images of immunofluorescence from liver of *Tlr4LoxTB* allele (*Tlr4*^{KO}) mice treated with 1x10^9 PFU Adenovirus serotype 5 expressing Cre and GFP with a strong tropism for hepatic tissue. 10 days after infection, mice were sacrificed and livers fixed in 4% PFA and stained for detection of GFP (green), and DAPI (blue). 7 Evenly spaced sections were quantified for % GFP expressing cells. Scale bar= 200um (p=0.0001). (**I**) Representative images of immunofluorescence from samples as in (H) were stained to detect GFP and F480, and relative quantification of percentage of F480 positive cells that are positive also for GFP (n/group=3). Scale bar=50um. (**J**) Plasma NEFA levels of RIP-DTR;*Tlr4*^{WT}, RIP-DTR;*Tlr4*^{WT};S100A9^{OE}, RIP-DTR;*Tlr4*^{KO};S100A9^{OE} , RIP-DTR;*Tlr4*^{Liver};S100A9^{OE} mice (n/group= 4-6) (p=0.053, p=0.0009, p=0.0025, p=0.0001) (**K**) Lipase activity in perigonadal fat of the aforementioned groups (n/group=3-4). Error bars represent SEM. Statistical analyses were done using one-way ANOVA (Tukey's post-hoc test) or 2 tailed student's t-test **H**, except for **G**, where mixed effects model was used. *P<0.05, **P<0.01, ***P<0.001, ****P<0.0001. Related to Figure 1.

Figure S2

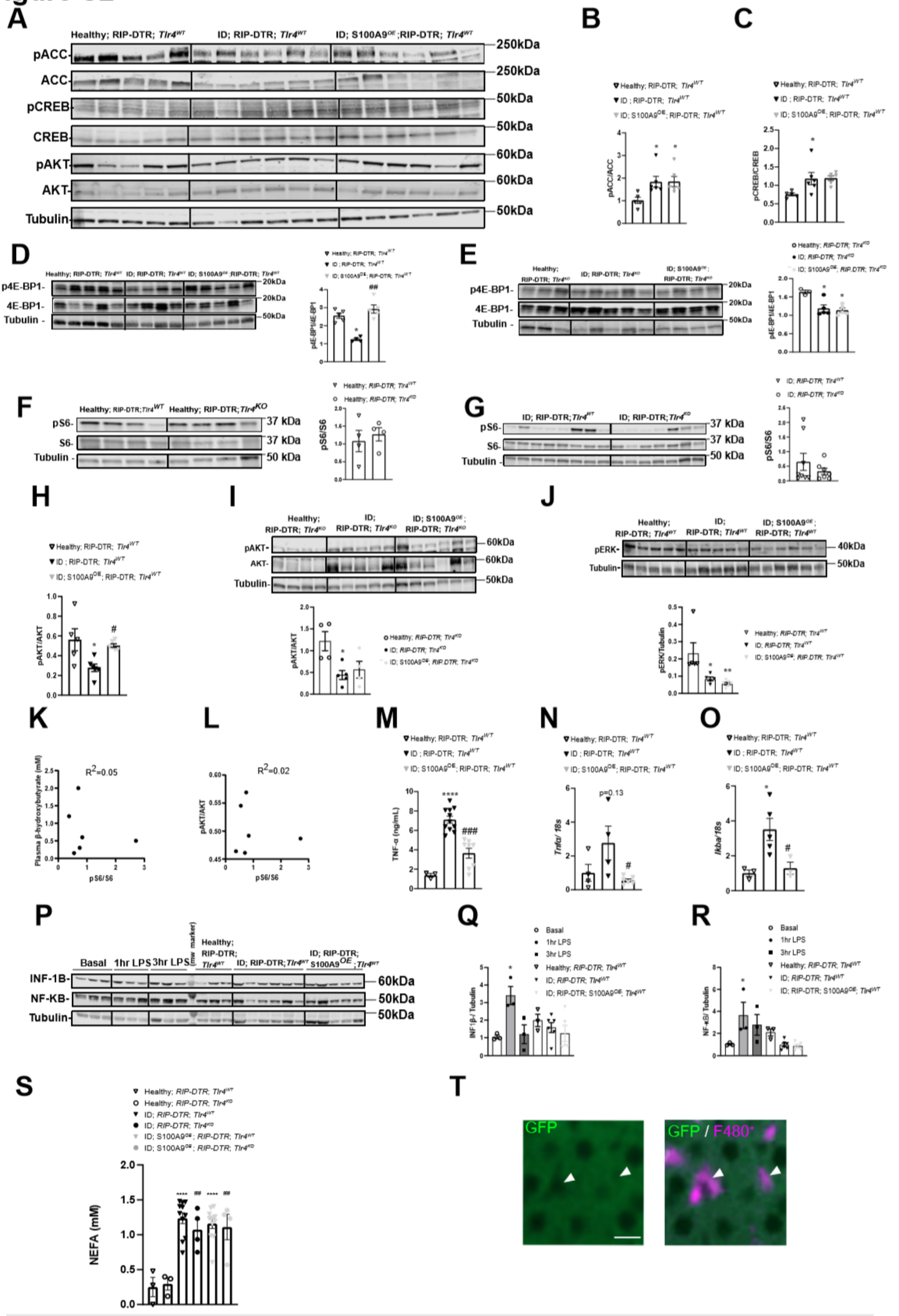
A
Healthy; RIP-DTR; Tlr4WT
ID; RIP-DTR; Tlr4WT
ID; S100A9OE;RIP-DTR; Tlr4WT
pACC
ACC
pCREB
CREB
pAKT
AKT
Tubulin
250kDa
250kDa
50kDa
50kDa
60kDa
60kDa
50kDa
B
pACC/ACC
C
pCREB/CREB
D
p4E-BP1
4E-BP1
Tubulin
20kDa
20kDa
50kDa
p4E-BP1/4E-BP1
E
Healthy; RIP-DTR; Tlr4KO
ID; RIP-DTR; Tlr4KO
ID; S100A9OE; RIP-DTR; Tlr4KO
F
pS6
S6
37 kDa
37 kDa
50 kDa
pS6/S6
G
H
pAKT/AKT
I
J
pERK
40kDa
pERK/Tubulin
K
R2=0.05
Plasma β-hydroxybutyrate (mM)
pS6/S6
L
R2=0.02
M
TNF-α (ng/mL)
N
p=0.13
Tnfa/ 18s
O
Ikba/18s
P
Basal
1hr LPS
3hr LPS
mw marker
INF-1B
NF-KB
60kDa
50kDa
50kDa
Q
INF1β-/ Tubulin
R
NF-κB/ Tubulin
S
NEFA (mM)
T
GFP
GFP / F480+

Supplemental figure 2. S100A9 overexpression does not affect hepatic AMPK or glucagon receptor signaling. (**A**) RIP-DTR; $Tlr4^{WT}$ mice were made insulin deficient (ID) with DT and underwent HTVI of either a plasmid encoding for mouse S100A9 (RIP-DTR; S100A9^{OE}) (n=6) or empty vector (n=6). 7 days into insulin deficiency liver lysates from these mice and healthy controls (n=5= were analyzed by for immunoblotting the indicated proteins and phosphorylation states. (**B-C**). Densitometry of immunoblot from (A) (p= 0.021 and p=0.03 for (B). p= 0,044 and p=0.055 for (C)). (**D**)Liver lysates from $Tlr4^{WT}$ healthy, ID or ID; S100A9^{OE} mice were analyzed for the indicated proteins and phosphorylation states (n/group= 5, 4 and 5) (p=0.0007, p=0.0001) (**E**). Liver lysates from $Tlr4^{KO}$ healthy, ID or ID; S100A9^{OE} mice were analyzed for the indicated proteins and phosphorylation states (n/group= 3, 5 and 4) (p=0.007, p=0.004). Healthy $Tlr4^{WT}$ (n= 4) and $Tlr4^{KO}$ mice (n=4) (**F**) and ID (**G**) $Tlr4^{WT}$ (n=8) and $Tlr4^{KO}$ (n=7) mice were analyzed by immunoblotting for the indicated proteins and phosphorylation states. (**H**) Densitometry of immunoblot from (A) (p=0.017, p=0.046). (**I**) Healthy, ID and S100A9^{OE} RIP-DTR; $Tlr4^{KO}$ mouse liver lysate was analyzed by immunoblotting the indicated proteins and phosphorylation states (n/group= 4, 5 and 6) (p=0.02). (**J**) Healthy, ID and S100A9^{OE} RIP-DTR; $Tlr4^{WT}$ mouse liver lysate was analyzed by immunoblotting the indicated proteins and phosphorylation states (n/group=5, 5 and 6) (p=0.02, p=0.004). Correlation analysis of hepatic pS6/S6 levels with plasma β-hydroxybutyrate levels (**K**) and pAKT/AKT levels (**L**). (**M**) Plasma samples from healthy, ID and ID; S100A9^{OE} mice was analyzed for TNFα levels by ELISA (n/group= 3, 11 and 8) (p=0.0001). qRT-PCR analysis of *Tnfα* (n/group= 4, 4 and 6) (**N**) and *IK-βα* (n/group= 3, 5 and 3) (p=0.04) (**O**) from livers of RIP-DTR healthy, ID or ID; S100A9^{OE} mice (p=0.028, 0.048). (**P**) Healthy RIP-DTR mice were treated with 10mg/kg i.p LPS and were sacrificed 1 and 3 hours after injection. Liver lysates were run alongside lysates from RIP-DTR healthy, ID or ID; S100A9^{OE} mice and analyzed for the indicated proteins (**Q-R**) Densitometry of blots from P (n/group=3, 3, 3, 3, 6 and 5) (p=0.022 (Q) (p=0.039 (R)). (**S**) Plasma Non-Esterified Fatty Acid (NEFA) levels of $Tlr4^{WT}$ and $Tlr4^{KO}$ RIP-DTR healthy, ID or ID; S100A9^{OE} mice (n/group = 3, 3,10, 4, 11 and 4) (p=0.001, p=0.005, p=0.0001, p=0.003). (**T**) $Tsc1^{fl/fl}$ mice were treated with 1x10^9 PFU of Adenovirus-- TBG-GFP. 10 days after injection mice were sacrificed and livers were fixed in 4% formaldehyde, embedded in paraffin and stained for GFP (green) HNF-4α and F4-80^{+} (magenta). Scale bar=50µm. Error bars represent SEM, statistical analyses were done using one way or two-way ANOVA (Tukey's post-hoc test) except for C where FDR was used *p≤ 0.05. For I and J correlation analysis was performed using Pearson's r. Western blot images shown in D-F and Pare cropped images. Source data are provided as a source data file. Related to Figures 2 and 3.

Figure S3

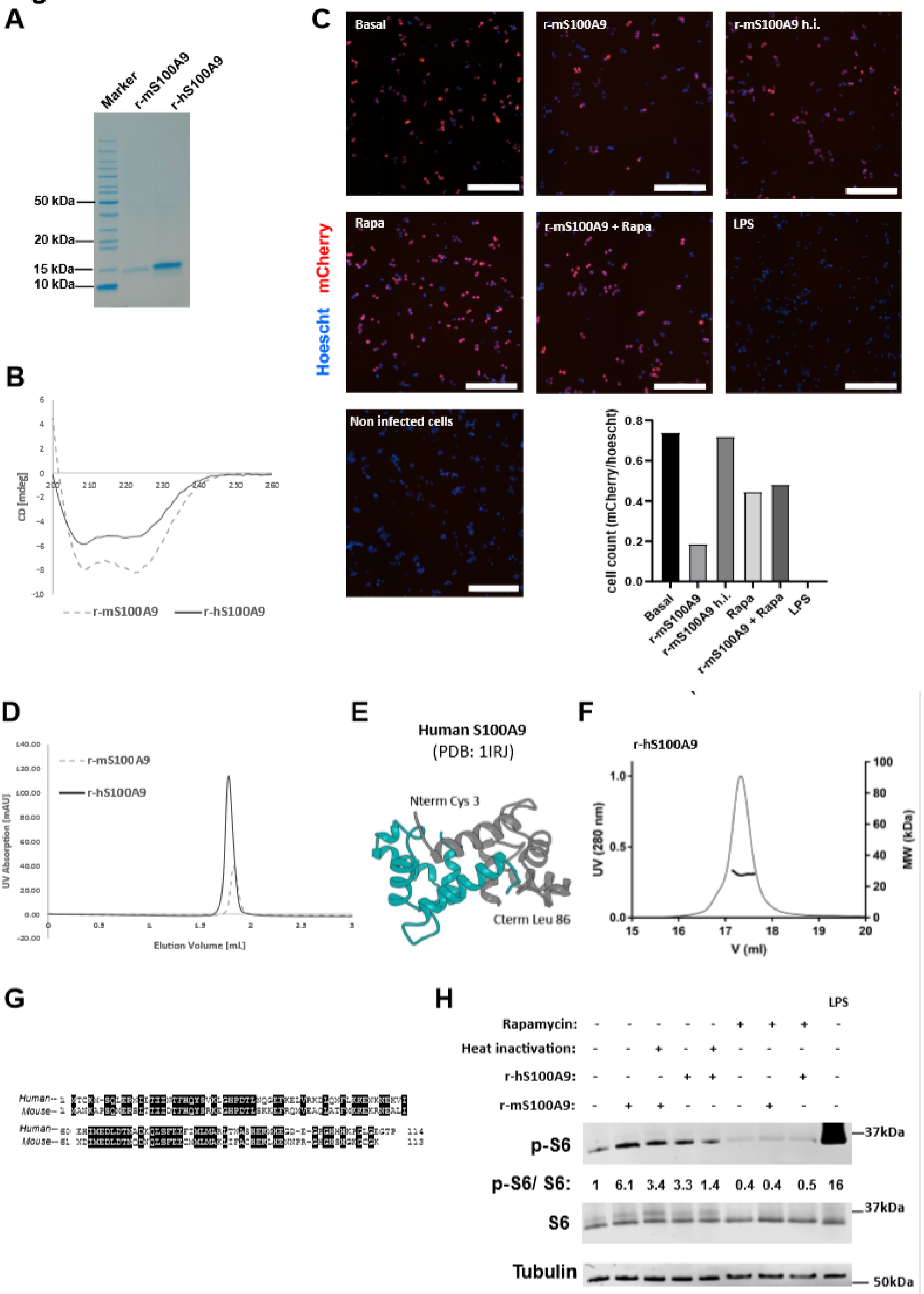
A
Marker
r-mS100A9
r-hS100A9
50 kDa
20 kDa
15 kDa
10 kDa
B
CD [mdeg]
r-mS100A9
r-hS100A9
C
Basal
r-mS100A9
r-mS100A9 h.i.
Rapa
r-mS100A9 + Rapa
LPS
Non infected cells
Hoescht
mCherry
cell count (mCherry/hoescht)
Basal
r-mS100A9
r-mS100A9 h.i.
Rapa
r-mS100A9 + Rapa
LPS
D
r-mS100A9
r-hS100A9
UV Absorption [mAU]
Elution Volume [mL]
E
Human S100A9
(PDB: 1IRJ)
Nterm Cys 3
Cterm Leu 86
F
r-hS100A9
UV (280 nm)
MW (kDa)
V (ml)
G
Human
Mouse
H
LPS
Rapamycin:
Heat inactivation:
r-hS100A9:
r-mS100A9:
p-S6
37kDa
p-S6/ S6:
1
6.1
3.4
3.3
1.4
0.4
0.4
0.5
16
S6
37kDa
Tubulin
50kDa

Supplemental Figure 3. Production of r-mS100A9 and r-hS100A9. (**A**) SDS page of r-mS100A9 and r-hS100A9. (**B**) Far UV circular dichroism spectra of r-hS100A9 (dark gray) and r-mS100A9 (dashed light gray) at roughly 5 μM concentration. (**C**) Representative image of immunofluorescence in RAW264.7 cells expressing mCherry-TOSI treated incubated with 2μg/mL of r-mS100A9 or r-hS100A9 (or their heat inactivated (h.i) forms) or 1μg/mL of lipopolysaccharides (LPS, from *E.coli* O111:B4, Sigma) for 4 hours, with or without 20uM Rapamycin . Quantification of mCherry positive cells / total cells is also shown. Scale bar= 100μm. n= 1x10^6 cells examined. (**D**) Analytical size-exclusion chromatography (SEC) using a Superose 6 Increase 3.2/300 column (Cytiva) of r-hS100A9 (dark gray) and of r-mS100A9 (dashed light gray), eluting at similar elution volumes of 1.78 mL and 1.83 mL, respectively. (**E**) Crystal structures of homodimeric S100A9 (cyan and gray). (F) Size-Exclusion-Chromatography Multi-Angle Light Scattering analysis of the r-hS100A9 showing a homodimer formation in solution. (**G**) Comparison of amino acid sequence between human and murine S100A9 (homology is highlighted in black). (**H**) THP-1 cells were incubated with 2μg/mL of r-mS100A9 or r-hS100A9 (or their heat inactivated controls) or 1μg/mL of lipopolysaccharide (LPS, from *E.coli* O111:B4, Sigma) for 4 hours with or without 20uM Rapamycin and cell lysates were analyzed by western blot. 1x10^6 cells were analyzed per condition. Related to Figure 4.

Figure S4

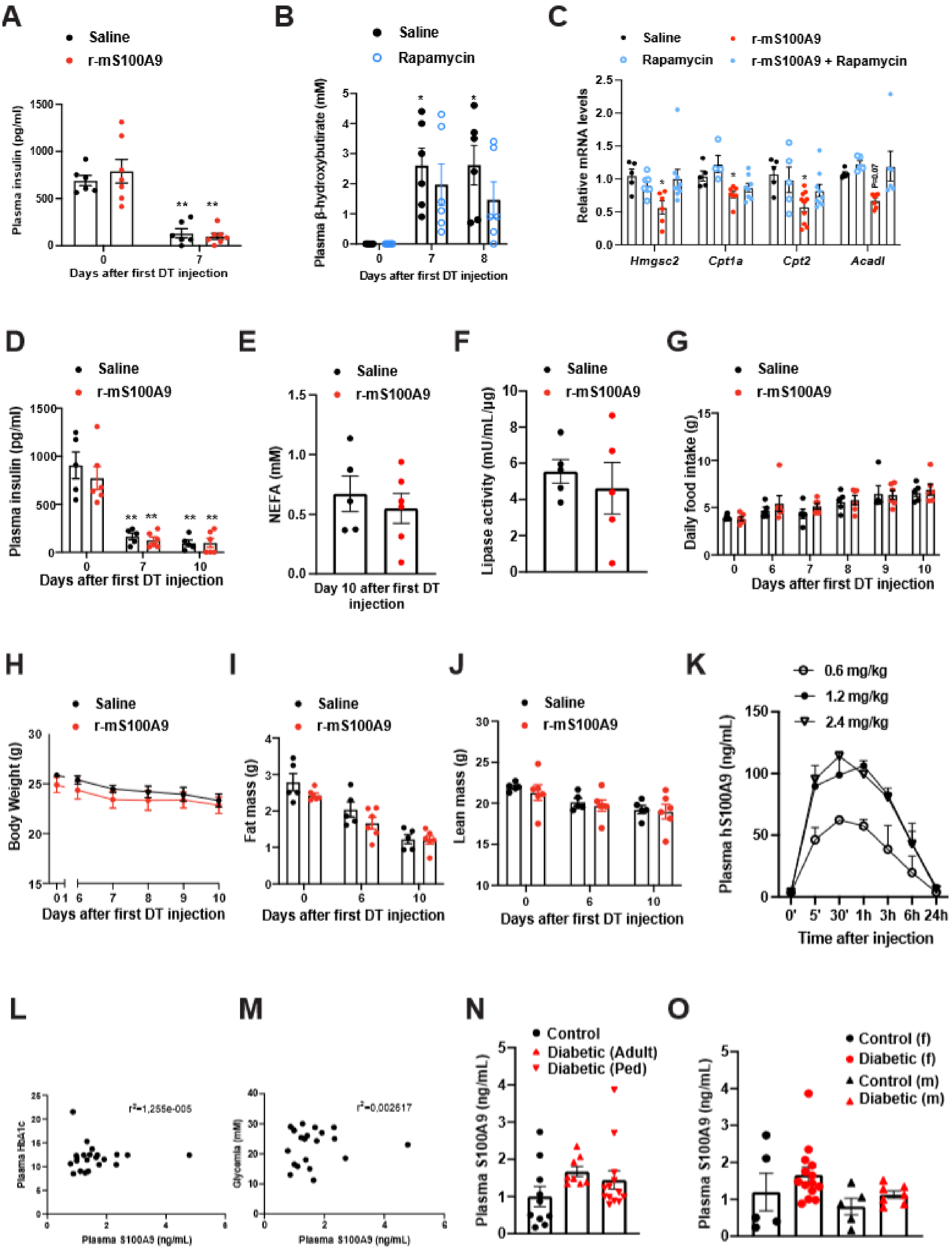

A
Saline
r-mS100A9
Plasma insulin (pg/ml)
Days after first DT injection
B
Saline
Rapamycin
Plasma β-hydroxybutirate (mM)
Days after first DT injection
C
Saline
r-mS100A9
Rapamycin
r-mS100A9 + Rapamycin
Relative mRNA levels
Hmgsc2
Cpt1a
Cpt2
Acadl
P=0.07
D
Saline
r-mS100A9
Plasma insulin (pg/ml)
Days after first DT injection
E
Saline
r-mS100A9
NEFA (mM)
Day 10 after first DT injection
F
Saline
r-mS100A9
Lipase activity (mU/mL/µg)
G
Saline
r-mS100A9
Daily food intake (g)
Days after first DT injection
H
Saline
r-mS100A9
Body Weight (g)
Days after first DT injection
I
Saline
r-mS100A9
Fat mass (g)
Days after first DT injection
J
Saline
r-mS100A9
Lean mass (g)
Days after first DT injection
K
0.6 mg/kg
1.2 mg/kg
2.4 mg/kg
Plasma hS100A9 (ng/mL)
0'
5'
30'
1h
3h
6h
24h
Time after injection
L
Plasma HbA1c
Plasma S100A9 (ng/mL)
M
Glycemia (mM)
Plasma S100A9 (ng/mL)
N
Control
Diabetic (Adult)
Diabetic (Ped)
Plasma S100A9 (ng/mL)
O
Control (f)
Diabetic (f)
Control (m)
Diabetic (m)
Plasma S100A9 (ng/mL)

Supplemental Figure 4. Safety of r-mS100A9 administration *in-vivo*. (**A**) Plasma insulin in DT treated mice at day 0 and 7 days after first DT injection (cohort indicated in Figure 5A) (n/group= 6, 7) (p=0.001). (**B**) RIP-DTR mice were rendered insulin deficient with DT and at day 8 were given intraperitoneal injections of either saline (n= 6) or 10mg/kg rapamycin (n= 6) and plasma β-hydroxybutyrate was measured (p=0.015, p=0.014). Metabolic assessments were done 3 hours after injection and food removal. (**C**) qRT-PCR analysis of genes from livers of ID RIP-DTR saline, rapamycin (10mg/kg), r-mS100A9 (0.6mg/kg), and rapamycin+r-mS100A9 treated mice (n/group= 5, 5, 9 and 8) (p=0.041, 0.049, 0.012). (**D**) Plasma insulin levels measured 0, 7 and 10 days after first DT injection (n/group=5, 6) (p=0.001). (**E**) Plasma NEFA levels (n/group=5, 6) and (**F**) adipose tissue lipase activity of r-mS100A9- and saline -injected control mice 7 days after first DT injection (n/group=5, 5). (**G**) Daily food intake, (**H**) body weight, (**I**) fat mass, and (**J**) lean mass of r-mS100A9- and saline -injected controls injection (cohort indicated in Figure 5G) (n/group=5, 6). (**K**) Plasma pharmacokinetic after subcutaneous injection of r-hS100A9 (n/group=2). (**L**) Correlation analysis of plasma S100A9 with HbA1c and (**M**) Glycemia (mM) in human diabetic patient samples (n=23). (**N**) Plasma S100A9 levels in diabetic adult (n=8) and pediatric (n=13) human patient samples and healthy controls. (**O**) Plasma S100A9 levels in male (m) and female (f) control and diabetic samples (n/group=5, 14 5, 7). Error bars represent SEM, statistical analyses were done using two-tailed t-test, or one way or two-way ANOVA (Tukey's post-hoc test) except in C where FDR was used. $^{**}p \leq 0.01$ and $^{***}p \leq 0.001$ showing comparison to basal. For L and M correlation analysis was performed using Pearson's r. Related to Figures 6 and 7.

Supplementary Table 1: List of sequence of primers used for real-time–qPCR. List of antibodies used for Western Blotting and Immunohistochemistry procedures.

Gene	Forward	Reverse
18s	ACCGCAGCTAGGAATAATGA	GCCTCAGTTCCGAAAACCA
Tlr4	CGCTGCCACCAGTTACAGAT	AGGAACTACCTCTATGCAGGG
Hmgcs2	ATACCACCAACGCCTGTTATGG	CAATGTCACCACAGACCACCAG
Cpt1a	CCATGAAGCCCTCAAACAGATC	ATCACACCCACCACCACGATA
Cpt1b	AGAAGTGTAGGACCAGCCCG	ACTTGCCTT TGTCCCGGAAAT
Cpt2	GGATAAACAGAATAAGCACACC	GAAGGAACAAAGCGGATGAG
Acadl	CTTGGGAAGAGCAAGCGTACT	CTGTTCTTTTGTGCCGTAATTCG
Tsc1	TCAAGCACCTCTTCTGCCTT	GTCACATGGCCTGGTTTCTT
IK-βα	CGGAGGACGGAGACTCGTT	CCATGGTCAGCGGCTTCT
TNFα	CCACGCTCTTCTGTCTACTGAACT	GATGAGAGGGAGGCCATTTG
	**Antibody**	
**Name**	**Supplier**	**Reference**
Phospho-S6 (Ser240/244	Cell Signaling Technology	5364
S6	Cell Signaling Technology	54D2
S100A9 (mouse)	Boster	PB9678
S100A9 (human)	Boster	PB9676
α-Tubulin	Proteintech	11224-1-AP
Na-K-ATPase	Cell Signaling Technology	3010
KDMI/LSD1	Abcam	ab140365
GFP	Abcam	ab13970
Phospho Acetyl CoA Carboxylase	Cell Signaling Technology	3661
Acetyl CoA Carboxylase	Cell Signaling Technology	3662
Phospho CREB	Cell Signaling Technology	9198
CREB	Cell Signaling Technology	9104
HNF-4α	Santa cruz	sc-374229
F4-80^{+}	Cell Signaling Technology	70076
mCherry	Thermo Fisher	M11217
Phospho 4E-BP1(Thr37/46)	Cell Signaling Technology	2855
4E-BP1	Cell Signaling Technology	9644

Supplementary Table 2: List of diabetic patient samples used for analysis**.**

**Patient ID#**	**Clinical Presentation**	**HbA1c**	**Adult/Ped.**	**Age range (Years)**<br>**$0 \leq$ a ≤ 10**<br>**$11 \leq$ b ≤ 25**<br>**$26 \leq$ c ≤ 50**<br>**d > 50**	**Sex**	**Sample Type**	**Glycemia (mM)**	**βHB (mM)**	**S100A9 (ng/mL)**
1	DKA (not inaugural)	15,3	Adult	c	M	plasma	25	12,6	1,3
2	DKA inaugural diabetes	10,4	Adult	c	F	plasma	24,3	7,3	1,7
3	DKA inaugural diabetes	11,5	Adult	c	F	plasma	27	9,3	1,9
4	DKA inaugural diabetes	9	Adult	b	F	plasma	18	6,8	1,4
5	DKA inaugural diabetes	12,3	Pediatric	b	F	serum	28	1,7	1,0
6	DKA inaugural diabetes	12,5	Pediatric	b	F	plasma	26	11,9	1,4
7	DKA inaugural diabetes	12,4	Adult	c	F	plasma	23	8,7	4,8
8	DKA inaugural diabetes	12,4	Pediatric	b	M	plasma	18,5	10,2	2,7
9	DKA inaugural diabetes	12,4	Pediatric	a	F	plasma	17,9	8,4	6,8
10	DKA inaugural diabetes	13,7	Adult	c	M	plasma	15	8,3	1,5
11	DKA inaugural diabetes	11,7	Pediatric	b	F	plasma	16,5	5,2	1,0
12	DKA inaugural diabetes	21,5	Pediatric	b	M	serum	13	2,3	0,9
13	DKA inaugural diabetes	11,9	Pediatric	a	F	plasma	11,2	5,8	1,6
14	DKA inaugural diabetes	8,7	Pediatric	a	M	plasma	30	11,4	1,3
15*	DKA (not inaugural)	12,5	Adult	b	F	plasma	25	11,6	2,3
16	DKA inaugural diabetes	8,5	Pediatric	a	F	serum	29	11,1	0,9
17	DKA inaugural diabetes	10,6	Pediatric	a	M	serum	21	2,7	0,8
18	DKA inaugural diabetes	12,4	Pediatric	a	F	plasma	25,4	11,4	1,2
19	DKA inaugural diabetes	11,2	Pediatric	a	M	serum	27,7	2,7	1,0
20	DKA inaugural diabetes	9	Pediatric	a	M	plasma	15,8	2,0	1,1
21	DKA (not inaugural)	12,4	Adult	c	F	plasma	28,8	13,1	1,7
22	DKA inaugural diabetes	11,4	Pediatric	a	F	plasma	25	9,0	1,3
23	DKA (not inaugural)	10,4	Adult	d	M	serum	28,8	12,3	2,3

* Average value of 4 repeated samples taken

Supplementary Table 3: List of control patient samples used for analysis

Sex	Age range (Years) 0 ≤ a ≤ 10 11 ≤ b ≤ 25 26 ≤ c ≤ 50 d > 50	Source	plasma/serum	βHB (mM)	S100A9 (ng/mL)
M	d	CTS	plasma	0	0,2
F	c	CTS	plasma	0,07	0,5
M	d	CTS	plasma	0	1,1
F	d	CTS	plasma	0	0,2
F	b	CTS	plasma	0	0,4
M	c	CTS	plasma	0	0,5
F	d	CTS	plasma	0	2,7
F	c	CTS	plasma	0	2,1
M	c	CTS	plasma	0	1,5
M	d	CTS	plasma	0	0,9
