# Supplementary material for: Hepatic non-parenchymal S100A9-TLR4-mTORC1 axis normalizes diabetic ketogenesis
Source: Nat Commun. 2022 Jul 15;13:4107. doi: 10.1038/s41467-022-31803-5 (PMC9287425; doi:10.1038/s41467-022-31803-5)
Supplement: Supplementary file 2 — Reporting Summary [file 41467_2022_31803_MOESM2_ESM.pdf]

nature portfolio

Corresponding author(s): Roberto Coppari
Ramadori Giorgio

Last updated by author(s): 8 June 2022

Reporting Summary

Nature Portfolio wishes to improve the reproducibility of the work that we publish. This form provides structure for consistency and transparency in reporting. For further information on Nature Portfolio policies, see our Editorial Policies and the Editorial Policy Checklist.

Statistics

For all statistical analyses, confirm that the following items are present in the figure legend, table legend, main text, or Methods section.

n/a	Confirmed	
☐	☒	The exact sample size (*n*) for each experimental group/condition, given as a discrete number and unit of measurement
☒	☐	A statement on whether measurements were taken from distinct samples or whether the same sample was measured repeatedly
☐	☒	The statistical test(s) used AND whether they are one- or two-sided<br>*Only common tests should be described solely by name; describe more complex techniques in the Methods section.*
☒	☐	A description of all covariates tested
☐	☒	A description of any assumptions or corrections, such as tests of normality and adjustment for multiple comparisons
☐	☒	A full description of the statistical parameters including central tendency (e.g. means) or other basic estimates (e.g. regression coefficient) AND variation (e.g. standard deviation) or associated estimates of uncertainty (e.g. confidence intervals)
☐	☒	For null hypothesis testing, the test statistic (e.g. *F*, *t*, *r*) with confidence intervals, effect sizes, degrees of freedom and *P* value noted<br>*Give P values as exact values whenever suitable.*
☒	☐	For Bayesian analysis, information on the choice of priors and Markov chain Monte Carlo settings
☒	☐	For hierarchical and complex designs, identification of the appropriate level for tests and full reporting of outcomes
☐	☒	Estimates of effect sizes (e.g. Cohen's *d*, Pearson's *r*), indicating how they were calculated

Our web collection on statistics for biologists contains articles on many of the points above.

Software and code

Policy information about availability of computer code

Data collection	Licor fluorescent detection , cytation
Data analysis	Gen5 image, Image J 1.53r, GraphPad Prism 9

For manuscripts utilizing custom algorithms or software that are central to the research but not yet described in published literature, software must be made available to editors and reviewers. We strongly encourage code deposition in a community repository (e.g. GitHub). See the Nature Portfolio guidelines for submitting code & software for further information.

Data

Policy information about availability of data

All manuscripts must include a data availability statement. This statement should provide the following information, where applicable:

- Accession codes, unique identifiers, or web links for publicly available datasets
- A description of any restrictions on data availability
- For clinical datasets or third party data, please ensure that the statement adheres to our policy

All source of the data are included in the main manuscript and supplemental information. The data supporting the findings of the current study that have not been deposited in a public repository are available from the corresponding author upon request. Source data are provided with this paper.

Field-specific reporting

Please select the one below that is the best fit for your research. If you are not sure, read the appropriate sections before making your selection.

☒ Life sciences ☐ Behavioural & social sciences ☐ Ecological, evolutionary & environmental sciences

For a reference copy of the document with all sections, see nature.com/documents/nr-reporting-summary-flat.pdf

Life sciences study design

All studies must disclose on these points even when the disclosure is negative.

Sample size	The number of animals per group was chosen based on previous analyses of similar studies (PMID: 31391467, 20855609). For experiments involving cell lines, the sample size was chosen based on previous analyses of similar studies (PMID: 32187554). For experiments involving human samples samples size was limited to the number of samples available at the Geneva universtiy hospital (HUG).
Data exclusions	No data were excluded from the analyses
Replication	We replicated the experiments for the number of times indicated in the manuscript.In all experiments, western blot and immunohistochemical analysis were repeated at least twice with similar results.
Randomization	Animals of similar genotype were randomly distributed into the different groups. In experiments involving cells, samples were randomly allocated to different treatments. For human studies, allocation to groups was not relevant because we received samples from diabetic and control patients from the Geneva universtiy hospital (HUG).
Blinding	blinding was not possible as the completion of the completion of the experiment required the investigators to know which were the experimental and control groups. However the data were analysed in an unbiased fashion.

Reporting for specific materials, systems and methods

We require information from authors about some types of materials, experimental systems and methods used in many studies. Here, indicate whether each material, system or method listed is relevant to your study. If you are not sure if a list item applies to your research, read the appropriate section before selecting a response.

Materials & experimental systems

n/a	Involved in the study
☐	☒ Antibodies
☐	☒ Eukaryotic cell lines
☒	☐ Palaeontology and archaeology
☐	☒ Animals and other organisms
☐	☒ Human research participants
☒	Clinical data
☒	☐ Dual use research of concern

Methods

n/a	Involved in the study
☒	☐ ChIP-seq
☒	☐ Flow cytometry
☒	☐ MRI-based neuroimaging

Antibodies

Antibodies used	The antibodies used in this work are described in methods section of the manuscript
Validation	• Anti-Phospho-S6 (Ser240/244) (Cell Signaling Technology, #5364) was validated in this manuscript by treating cells or mice with rapamycin (e.g., figures 4D and 5A).<br>• Anti-S6 (Cell Signaling Technology, #5364) was validated by Western blot by the manufacturer, showing a single specific band<br>• Anti-mouse S100A9 (Boster PB9678) was validated in this manuscript by treating murine cells with recombinant murine S100A9 (Figures 5A and C)<br>• Anti-human S100A9 (Boster PB9677) was validated in this manuscript by treating murine cells with recombinant humanS100A9 (Figures 5A and C)<br>• Anti-Na-K-ATP (Cell Signaling Technology, #3010) was validated in this manuscript by separation of cellular fractions (Figure 5C).<br>• Anti-KDMI/LSD1 (Abcam #140365) was validated in this manuscript by separation of cellular fractions (Figure 5C).<br>• Anti-GFP (Abcam #13970) was validated in this manuscript by treatment of mice with Adenoviral vectors expressing GFP (Figure S1H).<br>• Anti-pACC (Cell Signaling Technology, #3661) was validated by Western blot by the manufacturer, showing a single specific band<br>• Anti-ACC (Cell Signaling Technology, #3662) was validated by Western blot by the manufacturer, showing a single specific band<br>• Anti-pCreb (Cell Signaling Technology, #9198) was validated by Western blot by the manufacturer, showing a single specific band<br>• Anti-Creb (Cell Signaling Technology, #9104) was validated by Western blot by the manufacturer, showing a single specific band<br>• Anti-HNF-alpha (Santa Cruz, sc-374229) had a nuclear localization in hepatocytes (Figure S2R).<br>• Anti phospho 4E-BP1 (Cell Signaling Technology, #2855) was validated by Western blot by the manufacturer, showing a single specific band<br>• Anti 4E-BP1 (Cell Signaling Technology #9644) was validated by Western blot by the manufacturer, showing a single specific band

Eukaryotic cell lines

Policy information about cell lines

Cell line source(s)	ATCC (RAW 246.7 & THP-1)
Authentication	All cell lines are DNA fingerprinted
Mycoplasma contamination	All cell lines tested negative for mycoplasma
Commonly misidentified lines (See ICLAC register)	no commonly misidentified cell lines were used in the study

Animals and other organisms

Policy information about studies involving animals; ARRIVE guidelines recommended for reporting animal research

Laboratory animals	10-12 week old male RIP-DTR, TLR4 ^{LoxTB}, and TSC1 $^{fl/fl}$ mice on a mixed genetic background were used for this study. Details are also in the manuscript.
Wild animals	no wild animals were used in this study
Field-collected samples	No field collected samples were used in this study.
Ethics oversight	Animal studies were approved by the animal care and experimentation authorities of the canton of Geneva, Switzerland (animal protocol numbers GE/78/18, GE/207/19)

Note that full information on the approval of the study protocol must also be provided in the manuscript.

Clinical data

Policy information about clinical studies

All manuscripts should comply with the ICMJE guidelines for publication of clinical research and a completed CONSORT checklist must be included with all submissions.

Clinical trial registration	This clinical study was approved by Swissethics (BASEC ID: 2017-00470)
Study protocol	Plasmatic ketones and S100A9 content in healthy and decompensated diabetic subjects: providing evidence for, or against, the therapeutic use of S100A9 in the context of hyperketonemia.
Data collection	Blood samples were collected from healthy donors and decompensated diabetic people admitted to the Geneva University Hospital (HUG) before treatment aimed at correcting their ketoacidosis was initiated
Outcomes	Slight increase in plasmatic S100A9 level observed in poorly controlled diabetic subjects vs healthy subject
